# AAPM BTSC Report 377.B: Physicist brachytherapy training in 2022 – A survey of therapeutic medical physics residents

**DOI:** 10.1002/acm2.14501

**Published:** 2024-10-07

**Authors:** Samantha J. Simiele, Manik Aima, Christopher S. Melhus, Susan L. Richardson

**Affiliations:** ^1^ Department of Radiation Physics University of Texas MD Anderson Cancer Center Houston Texas USA; ^2^ Department of Radiation Oncology Stanford University Stanford California USA; ^3^ Department of Radiation Oncology Tufts University School of Medicine Boston Massachusetts USA; ^4^ Department of Radiation Oncology Swedish Medical Center Seattle Washington USA; ^5^ Present address: Department of Radiation Oncology University of Alabama at Birmingham Birmingham AL USA

**Keywords:** brachytherapy, education, residency, survey, training

## Abstract

**Background:**

A survey of medical physics residency program directors was conducted in Spring 2021 to examine the current state of brachytherapy (BT) training during residency. In this related work, a subsequent survey of therapeutic medical physics residents in 2022 was conducted to assess the confidence and experience of the trainees. Concerns for access to high‐quality and diverse training in BT have escalated in importance due to recent declines in BT utilization.

**Methods:**

A survey consisting of 26 questions was designed by a working unit of the Brachytherapy Subcommittee of the American Association of Physicists in Medicine (AAPM) and approved for distribution by the Executive Committee of the AAPM. The survey was distributed to current trainees and recent graduates of the Commission on Accreditation of Medical Physics Education Programs accredited therapeutic medical physics residency programs by the AAPM. The participant response was anonymously recorded in an online platform and subsequently analyzed using spreadsheet software.

**Results:**

The survey was distributed to 796 current medical physics residents or recent graduates over the course of 6 weeks in February and March of 2022. The survey received 736 views and a total of 182 responses were collected, with 165 respondents completing the survey in full. Among those responses, 110 had completed their residency training, with program start dates ranging from calendar years 2015 to 2021. Individual responses from the survey takers (including partial survey submissions) were evaluated and analyzed to compile results.

**Conclusions:**

Medical physics residents reported the highest levels of confidence and caseload for gynecological BT procedures when compared with other surveyed treatment techniques. This indicates opportunities to improve training and increase access to clinical caseload are needed in order to improve competency and confidence. Time constraints (clinical and rotation‐based) were indicated as impediments to BT proficiency. Medical physics residents reported enthusiasm for additional training opportunities in BT, and it is evident that additional structure and programs are required to ensure adequate access to BT training during residency.

## 1 INTRODUCTION

In a review of declining BT use in cervix and prostate cancer, Petereit et al. described brachytherapy (BT) as “often mistakenly viewed as an antiquated or even irrelevant modality, despite the central role it continues to play in the management of several cancer types.”^1^ For prostate cancer in the early 2000s, the authors described a 30%−48% drop in BT procedures depending on the type of clinic. Contemporary challenges attributed to this drop include increased surgery and IMRT use, changes in reimbursement rates, negative press, rigorous regulatory reporting requirements, and a lack of caseload and appropriate volume to train radiation oncology residents.

For cervical cancer, Han et al. reported a 25% drop in BT use for cervical cancer between 1988 and 2009 despite it being the standard of care.^2^ Similarly, Petereit et al. summarized the causes of the drop in the use of cervical BT to include “inadequate training” of residents due to caseload and “inadequate maintenance” of BT skills.^1^ Another study posited that the declining use of BT in cervical cancer was related to a lack of comfort and exposure to the technology despite the anticipated increase in mortality rate when not incorporating BT.^3^ The motivation for this work was based on the declining rate of use of BT in the United States^4^, ^5^, ^6^ and investigating if this decline was partially related to the education and training of medical physics residents.

As reported in a survey of medical physics residency program directors,^7^ the Brachytherapy Subcommittee (BTSC) of the Therapy Physics Committee (TPC) under the Science Council of the American Association of Physicists in Medicine (AAPM) began a comprehensive evaluation of the current state of BT training. To our knowledge, no report exists correlating confidence level and caseload for BT medical physics residents. A working unit was formed to facilitate the evaluation by performing surveys of the key stakeholders: the trainers (i.e., residency program directors) and the trainees (i.e., residents). This work presents the results of a survey that was distributed to the Commission on Accreditation of Medical Physics Education Programs (CAMPEP)‐accredited residency program trainees, to evaluate the experience of trainees in the areas of high dose rate (HDR), low dose rate (LDR), intraoperative radiation therapy (IORT), TransArterial Radioembolization (TARE), and radiopharmaceutical.

## 2 MATERIALS AND METHODS

### 2.1 Survey design

The survey consisted of 26 questions of four types including: select all that apply, multiple choice, five‐point scale, and free response. The questions were designed to collect information regarding six areas: (1) background information on the trainee and their training cohort (e.g., environment and duration of training); (2) the training methods employed for and experienced by the trainee; (3) the level of independence and related comfort with specific treatment modalities and anatomic site offered at their institution; (4) the future plans and employment interest of trainees and graduates; (5) the trainees interest in additional BT training opportunities; and (6) a BT‐related overview of contemporary trainee practice impressions. A copy of the approved resident survey is provided in Appendix A. Of note, the survey was designed in consultation with representatives of the Canadian Organization of Medical Physicists (COMP), the Australasian Brachytherapy Group (ABG), and the Groupe Européen de Curiethérapie and European SocieTy for Radiotherapy & Oncology (GEC‐ESTRO). The goal of this coordination was to produce a survey that could be administered by each group (see Section 5).

### 2.2 Approval process

The AAPM thoroughly vetted the survey content. The survey was first drafted and reviewed with representatives of COMP, ABG, and GEC‐ESTRO. Within the AAPM, the draft survey was then reviewed and approved by the BTSC before undergoing TPC review. The survey was subsequently distributed to both the Education and Professional Councils for comments and approval. Education Council presented the survey to the AAPM Executive Committee (ExCom), who approved the survey for distribution.

### 2.3 Survey distribution

AAPM staff adapted the survey for implementation in the QuestionPro online survey tool (QuestionPro Inc., Dallas, TX) and identified the recipient pool of medical physicists. The goal was to target current trainees and those who have completed training within approximately 5 years, including those who started training during the calendar years 2015−2021. A link to the survey was distributed to the identified trainees. The link was coded to each unique email address thereby ensuring integrity of the collected data. This list included 796 different trainees. The survey invitation was sent by AAPM headquarters staff, and a response was requested within 6 weeks. Reminder emails were sent at the completion of weeks 3 and 5 as well as a final reminder that was sent on the day of the survey deadline (March 2022).

### 2.4 Analysis

Numerical results were reviewed by the Working Group to identify inconsistencies and clarify analysis pools. Survey data were interpreted to present results for both the entire training cohort and those who had completed residency training. Numerical analysis, including summary statistics, was performed using Microsoft Excel (Microsoft, Redmond, WA). Free‐form text responses were interpreted using keywords and author interpretation. A statistical analysis was performed for Questions 11 and 12 using MATLAB (MathWorks, Natick, MA). Median and mode statistics were calculated for caseload and confidence levels reported by the survey participants. Given the ordinal nature of the data, Spearman Rho statistical test was deemed appropriate, a correlation coefficient was calculated to evaluate the correlation between the reported caseload and confidence level. “Corr” function was used in MATLAB to calculate the Spearman Rho coefficient with “Spearman” and “pairwise” options selected. The calculated coefficient values can have several qualitative interpretations. A pertinent interpretation described by Schober et al., defines the coefficient value of 0.00–0.10 as negligible correlation, 0.10–0.39 as weak correlation, 0.40–0.69 as moderate correlation, 0.70–0.89 as strong correlation, and 0.90–1.00 as very strong correlation.^8^ A correlation value greater than zero implies positive correlation and a value less than zero implies negative correlation.

## 3 RESULTS

### 3.1 Survey response rate

There were 182 responses from the survey recipients with 165 participants fully completing and submitting the survey and the remaining 17 participants partially completing the survey. This represents an overall response rate of 23%. The majority (96.7%) of respondents (176) attended/completed a residency in the United States of America and the rest in Canada. Most of the survey participants (110 out of 182; 60.4%) reported having graduated from their residencies while 72 reported currently attending a residency program. The average time to complete the survey was 17.9 min with a standard deviation (*k* = 1) of 24.3 min.

Many of the subsequent survey questions concern overall experience and confidence gained during residency. As such, the data and results that follow are representative of the 110 radiation oncology physics residents who reported completing a CAMPEP‐accredited residency and are thus labeled partial cohort [PC]. This population of graduated residents represents the confidence and gained experience of medical physicists entering the workforce. The remaining 72 respondents reported being in training at the time of the survey, and where appropriate, their responses are pooled with the graduates to represent the full cohort, noted as [FC]. For example, responses concerning infrastructure, staffing, and residency format questions include the FC, but questions regarding confidence reflect the opinions of residency graduates only to deduce the impact on the workforce. FC questions are designated with a “[FC]” and PC questions are designated with “[PC]” in Appendix A.

### 3.2 Summary of medical physics resident reported data

#### 3.2.1 Background information

Most graduated residents (94.4%) reported receiving 3 months or more of BT training with 38% receiving 3 months of training. The reported (FC) residency start dates amongst the participants were between 2015 and 2021 with a plurality (24.4%) of participants reporting 2020 as to when their residency began. 82% of the respondents reported that BT training was performed at their institution exclusively while the remainder reported partially or fully training at another institution.

#### 3.2.2 Training methods

There are several training methods used to teach BT‐related didactic material as reported by the graduates. 85.9% of the residents used independent review of the pertinent recommendations and regulations, 65.6% had assigned readings with structured meetings while 43.6% had assigned readings without structured meetings. 54.6% reported attending presentations from faculty/staff while 36.8% attended presentations from other residents during their BT training, and 25.2% reviewed AAPM virtual library presentations. 4.3% of the respondents received no didactic training.

As far as BT treatment planning is concerned, a majority of residents (73.6%) reported that at least two or more categories of personnel performed BT planning at their training institution. Physicists (90.2%), medical physics residents (55.8%), physicians (18.4%), dosimetrists (16.6%), and dedicated BT dosimetrists (9.8%) were listed as the BT planners. Observation (97.5%) and physicist instruction (94.5%) were reported as primary methods used to teach BT treatment planning to residents. The other methods reported were self‐learning through review of previous plans (68.1%), self‐learning using instructions (63.8%), instruction from a co‐resident (31.9%), online/electronic resources (23.9%), and dosimetrist instruction (21.5%).

Medical physics resident integration into the BT workflow is important for training. In the FC, 67% felt very integrated/involved, 19% were somewhat integrated/involved, 8% were neutral, and 6% were somewhat separated or very separated/removed.

#### 3.2.3 Caseload and confidence levels

Caseload and confidence information was collected as a function of treatment technique and body site, with respondents selecting the treatment technique available at their institution and then answering site‐specific questions included under that technique. In addition, all respondents were asked to summarize their caseload and confidence in six basic quality assurance activities generally applicable to BT. Respondents were instructed to “Check N/A only if your institution does not offer this procedure/technique/task.” It is possible that an institution performed a procedure (such as endobronchial) but the resident was not involved in these cases because the clinic's caseload was low and the resident did not have a chance to participate during residency. This is why an option of zero was made available.

Five general techniques were included in the survey and up to fourteen potential treatment sites could be assessed. For each treatment site, the respondent was asked about their caseload and confidence in treatment planning, treatment plan checking, and procedure participation. Table 1 outlines the techniques and sites that were included in the survey.

**TABLE 1:** Listing of treatment techniques and associated treatment sites incorporated in the caseload and confidence aspect of the survey. Data are reported for the total number of respondents that answered Questions 11 or 12.

Treatment technique	Treatment site/activity	No. responses
General quality assurance (QA)	Daily QA of remote afterloader	99 – Confidence, 100 – Caseload
	Source exchange QA of remote afterloader	100 – Confidence, 99 – Caseload
	Assay of loose seeds	100
	Connection of patient to afterloader	100
	Pre‐/post‐treatment surveys	100
	Applicator quality assurance	100
Low dose rate	Ocular/eye plaques	54
	Prostate	72
	Brain	16
	Interstitial—Other	37
High dose rate	Prostate	52
	Gynecological	97
	Endobronchial	26
	Skin/Surface	51
	Breast	51
	Interstitial/other	61
Intraoperative Radiation Therapy	Breast	22
	Gastrointestinal	18
TransArterial Radioembolization	Any site	20
Radiopharmaceuticals	Any site	22

**FIGURE 1:**
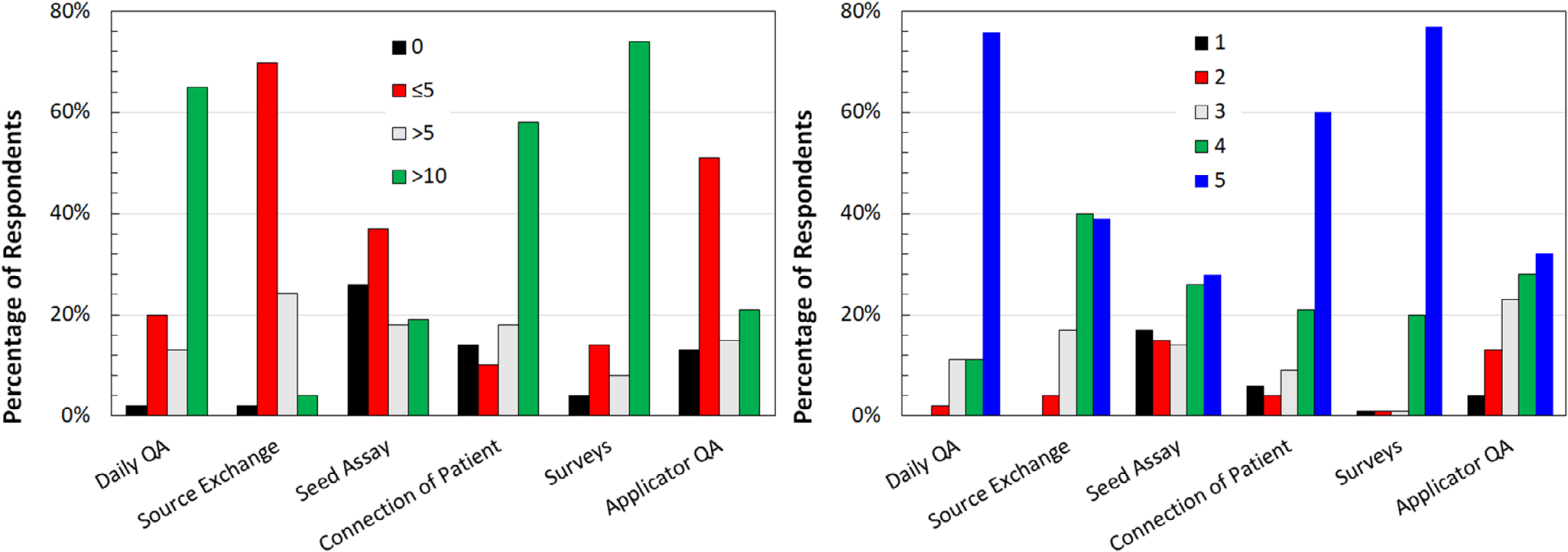
Reported caseload for regularly performed quality assurance activities (left panel) and related confidence level (right panel) with (1) representing “no confidence” and (5) representing “very confident” for graduated residents.

Figure 1 shows the caseload and confidence levels of the graduates for various quality assurance (QA) tasks. The reported percentages are normalized per question. Of note, for items that provided frequent opportunities for participation, for example, daily QA, patient connections, and patient surveys, the respondents noted high confidence levels. For activities that occurred with less frequency, for example, HDR source exchange, LDR source assays, and applicator QA, there may be fewer opportunities for training and respondents showed a corresponding lower distribution in confidence level. The results of the statistical analysis performed for this section are presented in Table 2. The Spearman Rho coefficient calculated using the reported data suggests a strong positive correlation (as defined in Section 2.4) between confidence levels and caseload for all the listed tasks except for the source exchange task (positive moderate correlation).

**TABLE 2:** Median and mode statistics for confidence level and caseload as well as Spearman Rho coefficient evaluating confidence level and caseload correlation for Question 11.

Competency (# of respondents)	Spearman Rho coefficient	Median confidence level	Mode confidence level	Median caseload	Mode caseload
Daily quality assurance of remote afterloader (99 – confidence, 100 – caseload)	0.7403	5	5	>10	>10
Source exchange quality assurance of remote afterloader (100 – confidence, 99 – caseload)	0.5472	4	4	≤5	≤5
Assay of loose seeds (100)	0.8133	4	5	≤5	≤5
Connection of patient to afterloader (100)	0.8060	5	5	>10	>10
Pre‐/post‐treatment surveys (100)	0.7528	5	5	>10	>10
Applicator quality assurance (100)	0.7450	4	5	≤5	≤5

**FIGURE 2:**
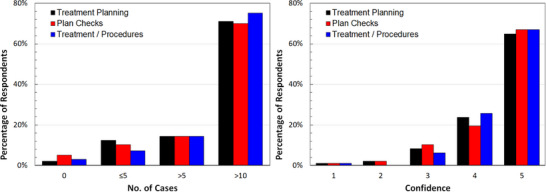
Reported caseload for HDR gynecological cases (left panel) and related confidence level (right panel) with (1) representing “no confidence” and (5) representing “very confident.”

Due to the large amount of data collected in the clinical portion of the survey, detailed results are available in Figure B1 and Table B1 in Appendix B (see Supplemental Material). As an illustration of this data, Figure 2 demonstrates the results for the treatment technique with the greatest reported caseload and confidence levels, i.e., that for high‐dose rate gynecological (GYN) treatment. For these treatments, high caseloads were reported (>70% of respondents participated in more than 10 cases) and these demonstrated a corresponding strong confidence level.

The median and mode values for GYN HDR treatment planning, plan checks, and treatment procedure with regard to confidence were 5 and 5; similarly, all three activities had median and mode caseloads of >10 and 10. The correlation coefficient between caseload and confidence level using Spearman Rho analysis was 0.72, 0.79, and 0.75 for treatment planning, treatment plan check, and treatment/procedure task, respectively. This suggests a strong positive correlation between caseload and confidence level for this procedure.

**FIGURE 3:**
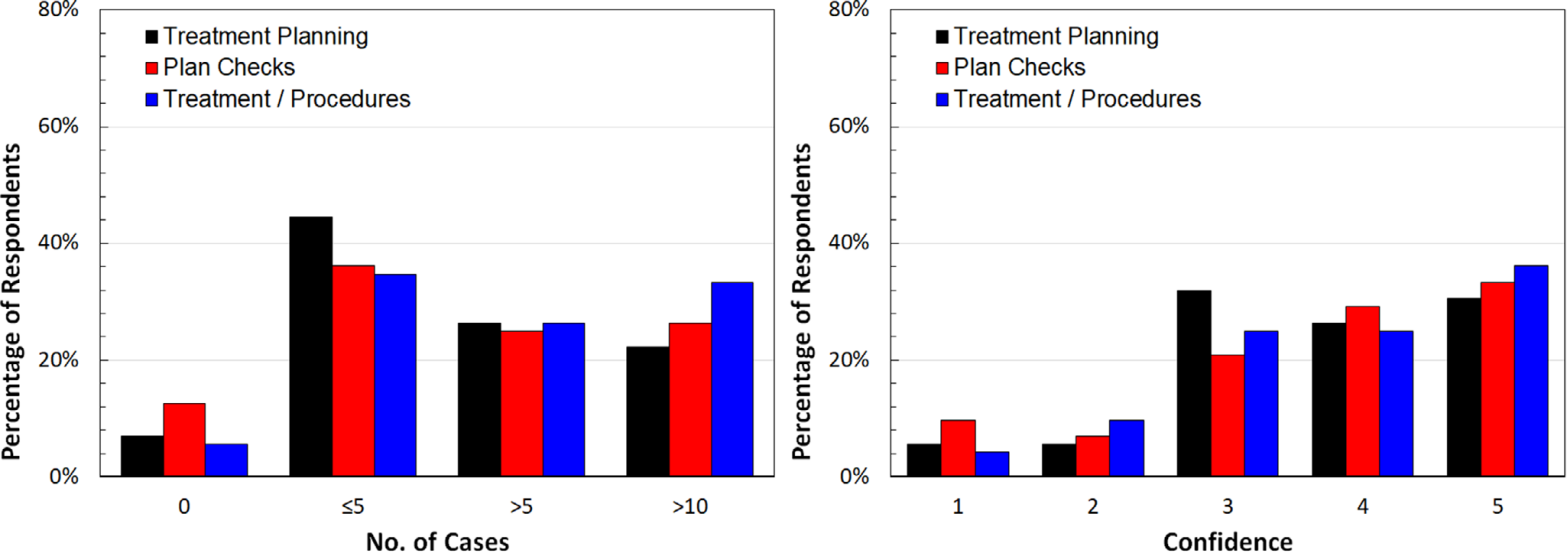
Reported caseload for LDR prostate cases (left panel) and related confidence level (right panel) with (1) representing “no confidence” and (5) representing “very confident.”

In contrast, Figure 3 shows the results for the LDR prostate technique, which was reported as the second most common modality by the program directors.^7^ Although there is a strong positive correlation between caseload and confidence level (>0.84 for all three tasks), the variability and suppressed confidence scores (refer to Appendix B, Table B1) to perform these tasks suggests that there might be a need for more learning opportunities required by the residents to become fully confident.

HDR GYN was the only modality and disease site combination with more than 50% of respondents indicating a confidence level of 5. For 10 out of the 14 modality and disease site combinations, most residents experienced ≤5 cases.

#### 3.2.4 Future employment and interest

**FIGURE 4:**
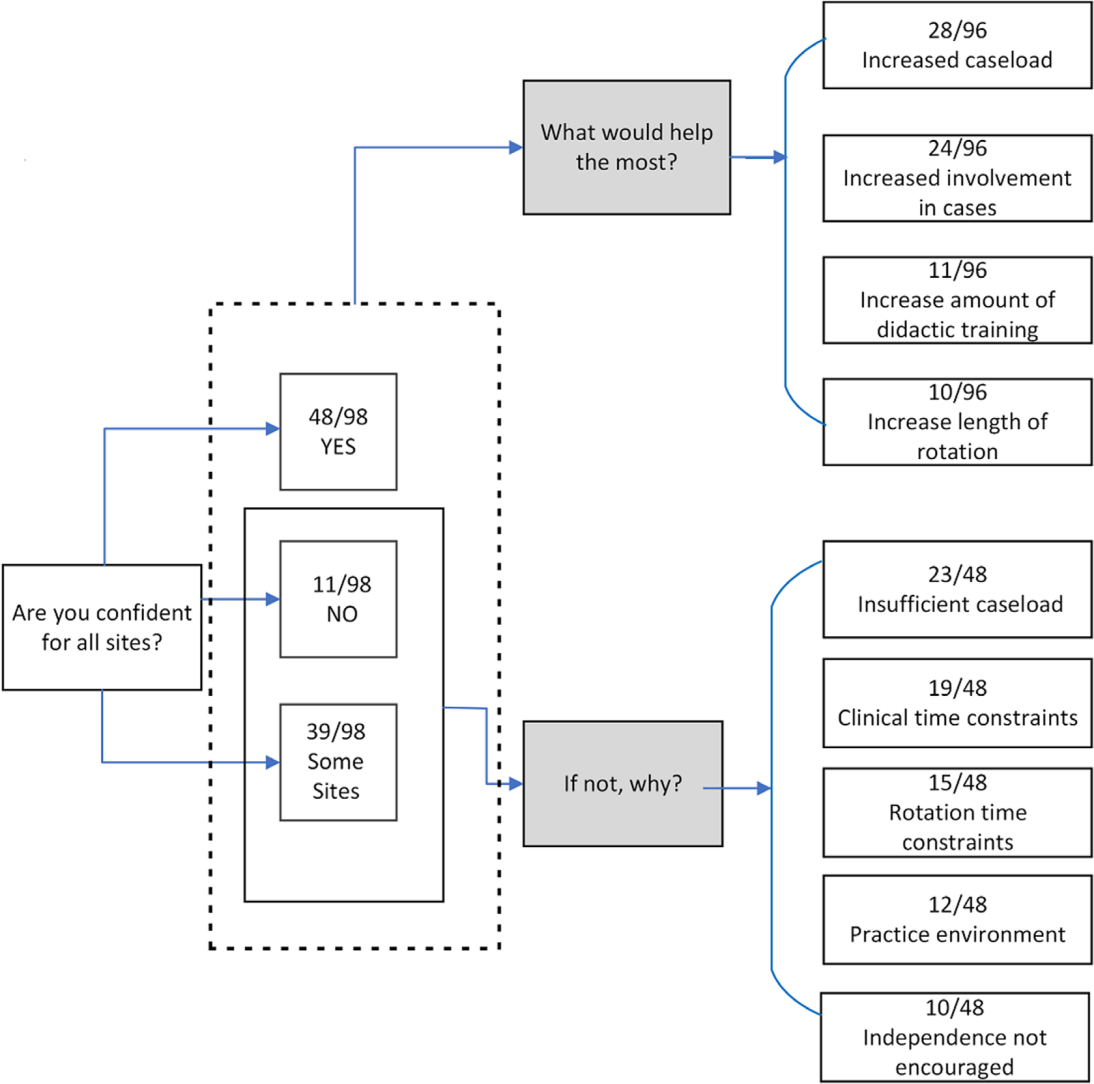
Confidence of graduated residents (PC) in performing brachytherapy procedures at the completion of their training. Distribution of reasons for not achieving confidence in performing procedures for all sites treated at their training institute as well as what factors would help the most in improving confidence are provided. Raw response values, including the number of respondents who selected an answer and the total number of respondents for a given question are provided. Some questions were “select all that apply.”

When asked if enough training was received during residency to practice BT confidently at the completion of their training program, 49% of respondents indicated they felt confident to perform procedures for all sites treated at their training institute, 39.8% indicated they felt confident to perform procedures for some sites, and 11.2% did not feel they gained enough experience to practice confidently for any BT treatment sites. Figure 4 provides the number of graduated residents (PC) who selected each option, the reason for their answer, and what change to training would help the most to improve confidence. The top five reasons provided by residents who did not achieve their desired level of training, defined as those individuals who did not indicate they felt confident to perform procedures for all sites treated at their training institute, included insufficient caseload (47.9%), clinical time constraints or the pace of the procedures (39.6%), rotation time constraints (31.3%), clinical environment (25.0%), and hesitancy of rotation supervisors to provide adequate independence (20.8%). When asked what factor would most help improve confidence levels, the most common response was an increase in caseload (29.2%) followed by increased involvement in cases (25.0%). Notably, increasing the amount of didactic training (11.5%) or the length of the rotation (10.4%) were not highly favored options among the respondents.

Most residents (69.8%) reported their interest level in performing BT procedures increased following the completion of their BT training. 25.0% were neutral, reporting no change in interest level and only 5.2% reported their interest level decreased following training completion. The majority (72.7%) want to perform BT at their first post‐residency position. 14.3% were unsure if they wanted their first position to include BT responsibilities, while 13.0% expressed a lack of interest in performing BT in future roles. When asked if their first position post‐residency would include BT responsibilities, 69.6% indicated they would be performing BT, while 4.3% were unsure, and 26.1% reported BT responsibilities would not be assigned.

#### 3.2.5 Additional BT training opportunities

Several questions were asked to gauge resident interest in receiving additional BT training opportunities and what type of training (e.g., elective rotations, rotations through other institutions, and/or fellowships) would be preferred.

**FIGURE 5:**
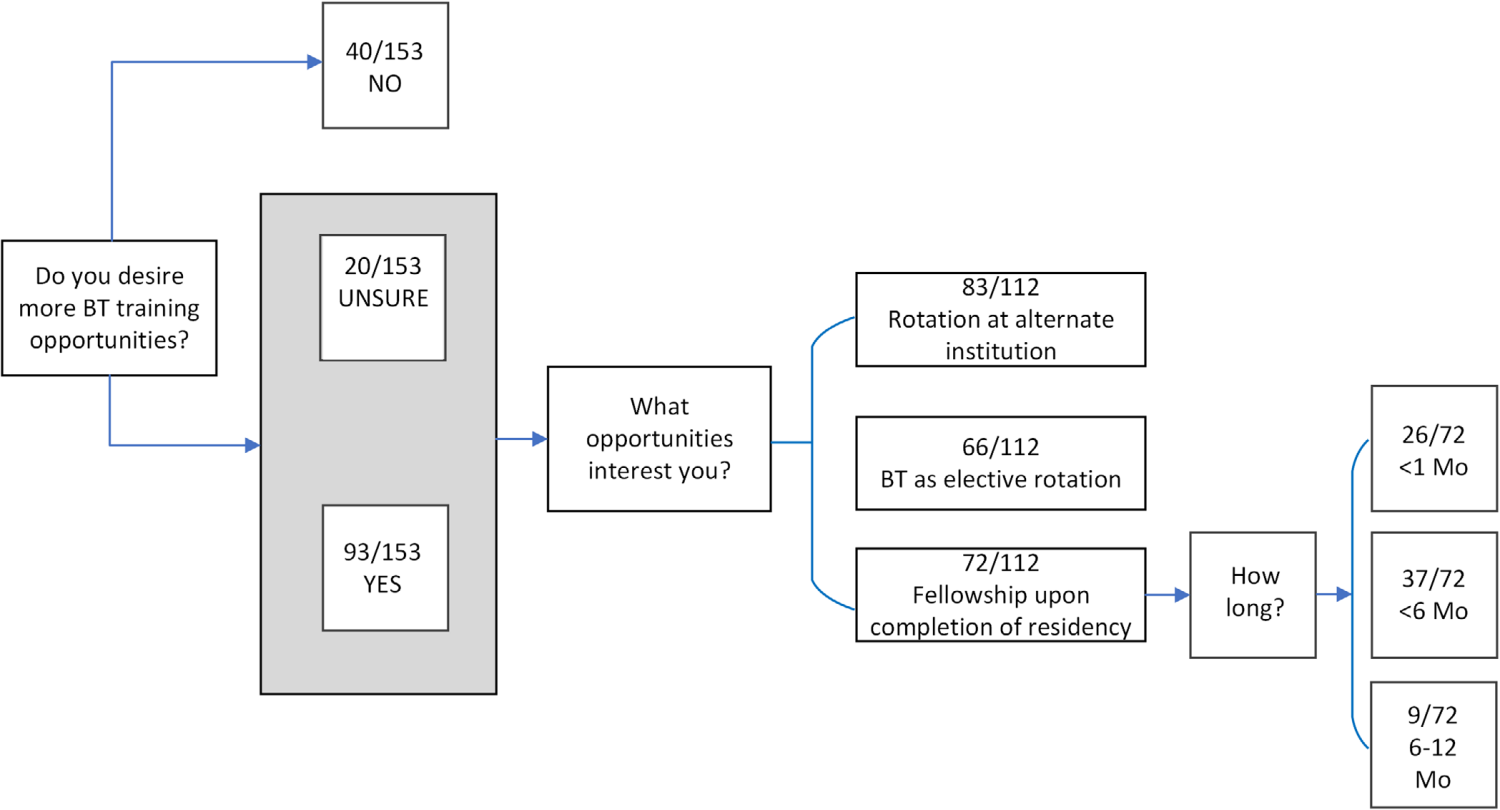
Interest level of current and graduated residents (FC) in receiving additional brachytherapy training opportunities. Raw response values, including the number of respondents who selected an answer and the total number of respondents for a given question are provided. Some questions were “select all that apply.”

The majority of residents (60.8%) desired more BT training opportunities. 13.1% were unsure and 26.1% did not want more training opportunities. Figure 5 presents the number of respondents (FC) who selected each response option. Of the respondents indicating they wanted or were unsure if they wanted additional training opportunities, 74.1% indicated interest in performing a BT rotation at an institution with a higher caseload or greater variety of cases than offered at their training location, 58.9% expressed interest in selecting BT as an elective rotation if one was offered, and 64.3% indicated interest in pursuing a fellowship in BT following the completion of training. The length of the proposed fellowship was important to the residents. Of those indicating interest in a fellowship, a slight majority (51.4%) were most interested in a fellowship less than 6 months in length, 36.1% preferred a fellowship less than 1 month in length, and 12.5% desired a fellowship ranging in length between 6 and 12 months.

#### 3.2.6 Contemporary trainee practice impressions

Several questions were asked regarding the residents' impression of BT practice and training at their institution. An overwhelming majority (90.8%) felt that their residency program directors valued BT education. This is an important metric in the ever‐expanding requirements and highly technical procedures that must be taught and learned over the course of residency.

**FIGURE 6:**
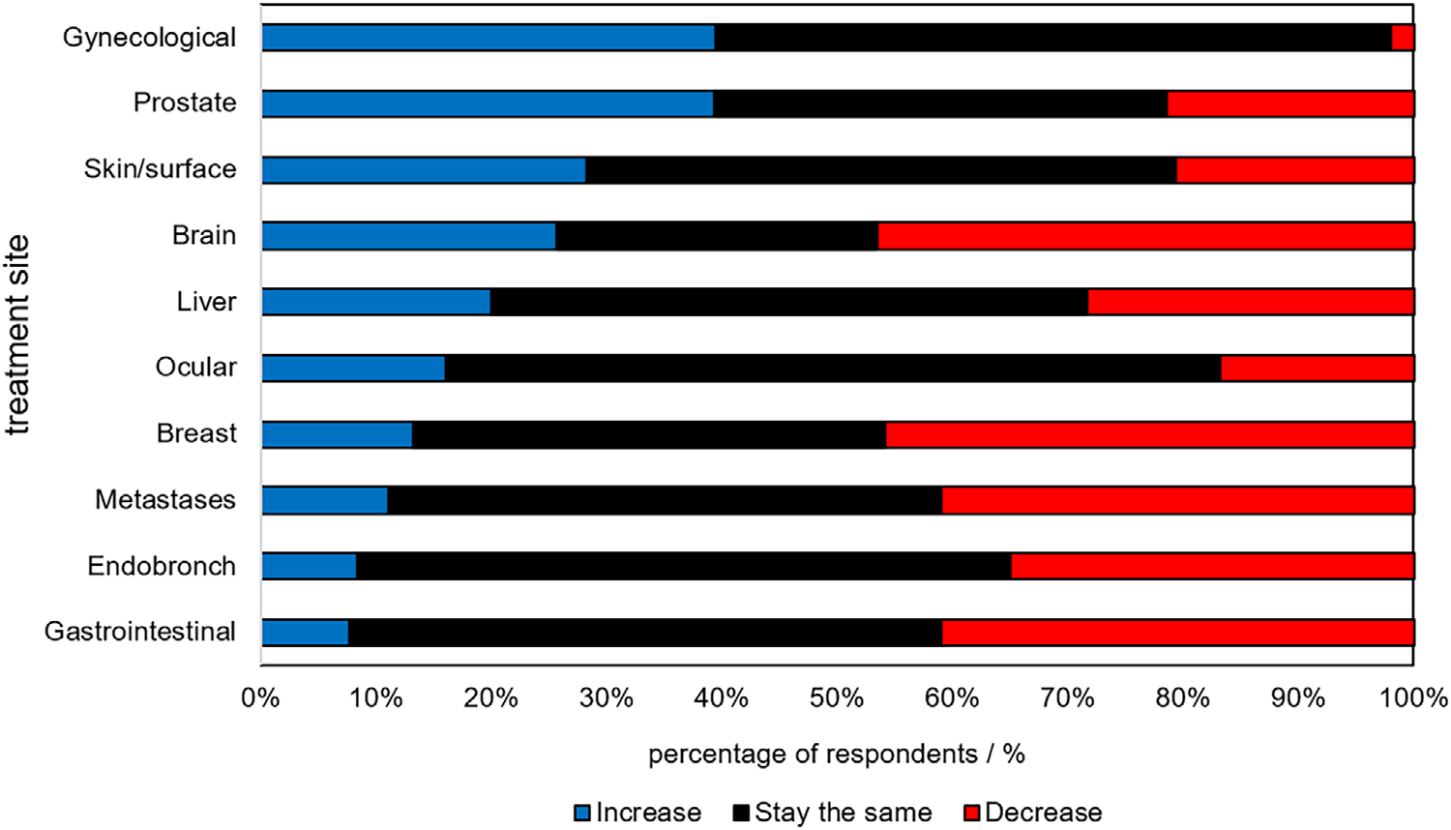
Impressions of BT practice trends by treatment sites reported by medical physics residency graduates. Results are the percentages of respondents who believe BT use will increase, decrease, or stay the same for different treatment sites.

In terms of site‐specific findings, residents typically felt the clinical relevance of using BT in each treatment site would stay the same over the next 10 years, with a few exceptions. First, almost half of respondents felt that breast BT relevance would decrease and only 13% believed it would increase. This may reflect the challenges, complexity, and time‐consuming nature of breast BT with comparative outcomes to external beam radiotherapy and surgery.^9^ In terms of gynecological BT, 59% believed the relevance would stay the same, and 40% believed it would increase. This positive result could reflect recent publications and presentations reiterating the imperativeness of BT treatments for overall survival for many gynecological cancers.^10^ Skin and superficial cancers are other treatment sites that were associated with the potential for increased BT utilization. Approximately a third (28.3%) of the respondents indicated a perceived increase and 51% perceived it would stay stable. New technological developments such as electronic BT and easier implementation of conical‐based HDR BT may increase patient treatment loads. A full summary of sites and indicated practice relevance is shown in Figure 6.

Regarding the importance and enjoyment of their BT rotation, respondents were asked if they felt it was important for medical physicists to learn BT. From a ranking scale of 1 (not important at all) to 5 (very important), 97% gave the rank of a 4 or 5, indicating BT is viewed as an important standard component of residency training. Regarding enjoyment, 42.1% of respondents answered that they enjoyed it more than their other rotations (rank = 5), with 34.9% indicating a level 4 response. While these results might suggest that BT is a “favorite” rotation amongst medical physicist residents, confirmation bias may be influencing these results.

#### 3.2.7 Feedback from survey participants

One “free response”‐type question was incorporated: “Is there additional information that you would like to provide regarding the BT component of your residency?” This question was posed to allow respondents to discuss issues and concepts that were not covered by other questions. Commonly reported themes and ideas from the responses were:

1. Lack of caseload and variety at their institution. Often, there would be enough of one type of procedure (e.g., HDR GYN) but not enough of others (e.g., HDR prostate). Some respondents discussed either wishing to travel to other institutions to watch cases or recalling the benefit of doing so, which provided them more confidence in rare(r) case presentations. An interesting anecdote from one respondent was that “see one, do one, teach one” is not sufficient for the complexity and difficulty of contemporary HDR BT cases.
2. Lack of support and time. Many respondents felt there was not enough time allowed to either see enough cases or be sufficiently involved in cases that were happening. One respondent indicated their director's interpretations of the regulations made it so they were not allowed to perform any direct patient activities, even under the supervision of a QMP. A few answers indicated that residents needed more personal integration into the workflow and felt stressed by the rotation. If a procedure had too many bystanders, respondents felt they were the first person to be removed from attending a case. Finally, some reported that the BT rotation was not prioritized by their institution, and thus, their training fell short.
3. Despite items 1 and 2, 75% of respondents stated they really enjoyed their BT rotations. Many felt their rotation was a success in part due to being part of a collaborative team. Some indicated high levels of confidence because they were allowed to perform work quasi‐independently. This sense of ownership and responsibility was indicated in helping the transition from resident to QMP at their next institution. More hands‐on training and incorporation into the workflow indicated a more successful BT rotation.

## 4 DISCUSSION

### 4.1 Comparison with other studies

Several comparisons are worth examining between the physics residents surveyed in this study and the radiation oncology residents surveyed in the work of Marcrom et al.^11^ Both cohorts had a similar response rate of 22.9% for medical physicists and 25.6% (145/567) for radiation oncologists. The data reported by Marcrom et al. was limited to radiation oncologists who were in their PGY4 or PGY5 years of training. The majority of each group believed their program directors valued resident education in BT and that it was important for residents to learn BT during their training.

Comparing the physics resident response rate to the radiation oncology resident response rate in Marcrom et al., the residents concurred that BT use will either increase or stay the same over the next 10 years for all sites, generally within a few percent except for breast BT. The comparison between these datasets is shown in Table 3.

**TABLE 3:** Percentage of residents who believe BT use will either increase or stay the same over the next 10 years.

	Prostate	GYN^a^	Breast	Skin
Medical residents	78	95	66	78
Physics residents	76.6	98.0	54.2	79.3

^a^ Marcrom et al. asked about cervix and endometrium separately, which are combined in this presentation under GYN.

Interest was expressed by both groups for receiving additional BT training, although the preference for duration and format of training varied considerably between the physicists and physicians. While medical physics residents’ interest in additional training was relatively uniform across the various training options, the radiation oncology residents demonstrated a preference for on‐the‐job training (90%), a short 1–2‐week American Brachytherapy Society (ABS) sponsored observership (49%), and only 2.2% in a formal fellowship. In contrast, the majority of medical physics residents expressed interest in completing a BT fellowship (64.3%), performing a BT rotation at another institution with a higher caseload (74.1%), or selecting BT as an elective rotation during residency (58.9%).

Data collected from the medical physics trainees and the program directors were compared.^7^ The majority of trainees (94.4%) and directors (86.4%) agreed that at least 3 months of time was spent learning BT during residency. Both cohorts believed BT education was valued during residency, with the majority of trainees (90.8%) believing their program director valued their BT education, and 97.7% of program directors reporting it was important or extremely important for residents to learn BT. The majority of directors (96.4%) also indicated BT is an essential modality in radiation therapy. Both groups agreed that resident confidence and ability at the end of training could be improved.

Approximately half (49%) of trainees reported feeling confident at the end of their residency to treat all sites treated at their training institute and (52.8%) of directors believed residency sufficiently prepared physicists to practice BT independently at the completion of training. Both groups agreed BT fellowships could be valuable, with 64.3% of residents expressing interest in completing a fellowship and 87.3% of directors believing their residents “could” or “could possibly” benefit from completing a fellowship.

Residents reported observation (97.5%) and physicist instruction (94.5%) as the primary methods used by their programs for teaching treatment planning. Alternative teaching approaches may be warranted given program directors reported treatment planning being the area in which the fewest residents achieved independence. Many options exist, and some would be simple to implement. Examples include the development of internal anonymized scan databases for residents to practice treatment planning, the generation of step‐by‐step instructions for residents to follow, and the implementation of a competency test to confirm resident ability. On a larger scale, organizations could provide and record webinars of the treatment planning process for resident access and review.

One area of discordance between residents and program directors is the percentage of residents who perform BT in their first post‐training position. Approximately two‐thirds (66.7%) of graduated residents reported they would perform BT in their first position. In our first study, only 17% of directors anticipated this fraction of their residents would be performing BT immediately following training. One‐third (32.1%) of directors were unsure whether their residents would perform BT in their first position. Given the majority of physicists expect to be responsible for BT procedures in their first position, the importance of achieving confidence in BT during residency cannot be overstated. The uncertainty of the directors in whether their trainees continue to perform BT could be due (in part) to the current process of achieving AMP status at the time of board certification. The upcoming changes to this process will require program directors to decide if they will attest to AMP status at the end of the residency, a consideration previously discussed by Aima et al.^7^

### 4.2 Challenges in BT training

Gill et al. summarized their review with the recommendation that physicians should “participate in educational programs to increase exposure” to BT.^3^ In a survey of chief residents, Nabavizadeh et al., found that 40−85% of residents reported inadequate exposure to both HDR and LDR BT depending on treatment site.^12^

While these studies focus on physicians and physician residents, it is important to acknowledge the training and education of other members of the team, including medical physicists and medical physicist residents. While the ABS “300 in 10” initiative aims to improve physician training,^13^ there is no similar fellowship or initiative for physicists. As described in this report, inadequate training due to caseload and time constraints will affect the future availability of competently trained BT medical physicists.

Additional training opportunities for radiation oncology residents are readily available; however, opportunities for medical physics residents are not. The absence of additional training opportunities for medical physics residents may be a contributing factor to the relatively high level of interest expressed by physics trainees in pursuing additional opportunities. As discussed in Section 3.2.5, physics residents are eager and willing participants in further BT training opportunities.

The subcategory of radiopharmaceutical administration is an interesting aspect of BT training. Both therapy and nuclear medicine trainees may be exposed to radiopharmaceutical administration, and/or may be expected to perform these procedures in their future positions. While technically a sealed source, TARE is included in this discussion due to similarities in administration. AAPM Report 249 indicates Y‐90 microsphere treatment as an optional training topical item, while there are no specific mentions of radiopharmaceutical administrations, including those that are relatively new and gaining popularity, such as treatments using radionuclides of Lu‐177 and Ra‐223. Our survey found that only 27% of respondents performed TARE at their institution, and only 25% did other radiopharmaceuticals (Table 1). For TARE, the most common caseload found was ≤5 and the confidence levels were around 3 or 4, depending on the task. For radiopharmaceutical sites, the results were similar, with confidence levels being slightly higher. This is unsurprising based on the relatively simple dosimetry, i.e., associated with uniform dosing prescriptions for radiopharmaceuticals. The main issue with these treatments was the few number of respondents, suggesting a lack of exposure at the training level despite increased adoption in the clinic. In terms of attestation (to be covered in the following section), the requirements for an AMP performing these tasks are undefined as there is no guidance from the NRC regarding the training and experience requirements for unsealed byproduct material or for low dose‐rate sources (e.g., Y‐90). The ACR technical standards reference states that an AMP in therapy or diagnostic subfields is qualified with appropriate, procedure‐specific training.^14^

### 4.3 Attestation of Authorized Medical Physicists

As noted in AAPM BTSC Report 377, over 40% of Medical Physics Residency Program Directors responding to a 2021 survey did not attest to Authorized Medical Physicist (AMP) eligibility for graduating residents, as stipulated on NRC form 313A(AMP).^15^ For these graduates, the AMP‐eligibility attestation may be essential to practice in specific clinical roles involving radionuclide sources. Without this attestation, an AMP‐candidate would need to work under the supervision of another AMP until the required training durations have been met, namely 1 year of full‐time training experience and 1 year of full‐time work experience. Starting in 2007, the American Board of Radiology (ABR) associated medical physicist board certification with AMP eligibility and included the text “AMP eligible” in writing on diplomates certificates.^16^ However, the ABR has announced intentions to cease this practice at the end of 2023, and the Nuclear Regulatory Commission (NRC) has announced intentions to remove the ABR from the list of specialty board certifications that meet, by definition, the training requirements for AMP status. For medical physicists receiving certification by the ABR after 2023, the only pathway to AMP status will be to submit training records and documentation—including the attestation of preceptors—directly to the NRC or the appropriate agency within an agreement state.

While the ABR has only provided AMP‐eligible designation for 15 years, these expected changes will have clear and immediate impacts on the emerging medical physics workforce starting in 2024. Smaller employers may be less likely to hire new graduates due to the training and oversight needed to achieve AMP status, especially if residency program directors are not attesting to the training and experience of resident graduates. As noted in Section 3.2.4, 72.7% of graduating residents participating in this survey expressed an interest in practicing BT in their first position following residency. This strong interest, expressed by the emerging workforce, may be challenged in achieving AMP status in light of the lack of attestation and the forthcoming changes expected by the ABR and NRC regarding the relationship between certification and AMP eligibility.

### 4.4 Limitations of this work

Limitations of the work include the standard drawbacks of using surveys to gather data. These include a nonresponse bias, i.e., survey takers may be more inclined to answer survey questions regarding BT if they felt strongly about the topic or their rotations (positively or negatively), and recall bias, i.e., respondents who had completed their residency at the time of the survey could inaccurately remember their experience. Respondents in this survey were also required to self‐evaluate their competency, which may or may not be an accurate assessment of their clinical skills.

Also, many survey takers may have experienced training during the SARS CoV‐2 pandemic, which has been shown to “[decrease] clinical experience, [reduce] case volume, and [disrupt] educational activities” during residency.^17^ This, in turn, could lead to less success in mastery of BT techniques and processes. Finally, the data collected for confidence levels and caseload for Questions 11 and 12 was ordinal in nature to facilitate quicker responses and potentially increase the number of survey respondents (no tedious individual entries). Consequently, the data type precludes the calculation of confidence intervals for caseload in association with confidence levels.

### 4.5 Survey impressions

While the purpose of this survey was to gather information regarding the caseload and confidence levels of BT trainees, some general impressions of the state‐of‐training can be summarized from the data. These are not recommendations, but themes that arose from the analysis of the data from both the program director and resident surveys.

- Caseload, which was identified as a significant issue in both surveys, is not high enough to create confident trainees except in a few clinical sites such as GYN HDR. Prior to 2022, five cases were required for physicians in specified anatomical sites to meet Accreditation Council for Graduate Medical Education (ACGME) requirements. However, physicists performing greater than five cases did not necessarily result in confidence in the procedure according to our survey. The number of cases required to gain confidence is not readily apparent, but in some cases may be greater than 5. The ACGME case requirements for physicians were updated to seven interstitial and 15 intracavitary BT procedures in 2022.^18^
- The majority of training programs are unable to provide a multitude of BT clinical experiences to trainees. Achieving greater breadth in training may require cross‐institutional coordination. For example, radiopharmaceuticals that are offered at only a few institutions.
- Fellowships and/or internships would be welcomed according to the survey respondents. This could help with the first two items articulated: number and variety of cases. While this practice is being utilized in physician residency programs and training, this happens rarely and, on an ad‐hoc basis for physicists.
- Medical physicist comfort levels in general, were comparatively less than the physicians, as found by Marcrom et al.,^11^ while the anticipated future role of BT was found to be similar between physicians and physicists.

## 5 FUTURE WORK

This work represents the current status of BT training through the lens of current and recent graduates of CAMPEP‐accredited residents. The survey described above was designed to also gather data about the BT training received internationally in Australasia, Canada, and Europe. Future work will evaluate and compare the BT training experiences of AAPM‐affiliated medical physics residents with those affiliated with ABG, COMP, and GEC‐ESTRO.

## 6 CONCLUSIONS

There are significant challenges inherent in the current system of preparing medical physics trainees for clinical practice. A survey of self‐reported confidence and competence identified that medical physics residents typically achieved success and mastery only for gynecological BT procedures, with a notable concentration in vaginal cylinder‐based treatment, based on the results of this work and those in Report 377.^7^ However, nearly every other treatment technique and/or treatment site indicated opportunities to improve training and increase access to clinical cases in order to improve competency and confidence. Impediments to gaining BT proficiency included time constraints (clinical and rotation‐based) and caseloads, that is, opportunities to gain experience in specific clinical situations. Fortunately, medical physics residents reported enthusiasm for additional training opportunities in BT, but training opportunities remain limited and current program architectures may not be optimized to maximize learning opportunities. A societal effort is needed to examine this issue and ensure access to high‐quality BT training. This ensures the continuity of an essential treatment modality while prioritizing patient safety.

## AUTHOR CONTRIBUTIONS

All authors worked on all the aspects of the manuscript. Samantha J. Simiele and Manik Aima contributed equally to the publication and should be considered joint first authors. Two authors acted as more senior authors in a mentorship arrangement. The survey design, results, and written summary were all contributed by all authors.

## CONFLICT OF INTEREST STATEMENT

The Chair of the BTSC Unit 72 has reviewed the required Conflict of Interest statement on file for each member of BTSC Unit 72 and determined that disclosure of potential Conflicts of Interest is an adequate management plan. The members of BTSC Unit 72 listed below attest that they have no potential Conflicts of Interest related to the subject matter or materials presented in this document. Samantha J. Simiele, PhD; Manik Aima, PhD; and Christopher S. Melhus, PhD. The members of BTSC Unit 72 listed below disclose the following potential Conflict(s) of Interest related to subject matter or materials presented in this document. Susan L. Richardson, PhD—Deputy Editor of the JACMP.

## Supporting information

Supporting information

## APPENDIX A BRACHYTHERAPY TRAINING SURVEY: MEDICAL PHYSICS RESIDENTS

### A.1

(Includes Survey Logic)

**FC = Full Cohort**

**PC = Partial Cohort**

1. Which professional society contacted you regarding this survey? [FC]
  ⚬ AAPM
  ⚬ ABG
  ⚬ COMP
  ⚬ ESTRO
2. What region was/is your training in? [FC]
  ⚬ North America
  ⚬ Australasia
  ⚬ Europe
  **Table:**
  Skip Logic:	
  North America:	**If selected, jump to** 4. [Q3.2a] 3. What country was/is your training in?
  Australasia:	**If selected, jump to** 5. [Q3.2b] 3. What country was/is your training in?
  Europe:	**If selected, jump to** 6. [Q3.2c] 3. What country was/is your training in?
3. What country was/is your training in? [FC]
  ⚬ Canada
  ⚬ United States
  **Table:**
  Show/Hide Question
  Default > Hide Question
  **If Criteria** Q2a **is met show question**
3. What country was/is your training in? [FC]
  ⚬ Australia
  ⚬ New Zealand
  **Table:**
  Show/Hide Question
  Default > Hide Question
  **If Criteria** Q2b **is met show question**
3. What country was/is your training in? [FC]
  ⚬ Austria
  ⚬ Belgium
  ⚬ Bulgaria
  ⚬ Croatia
  ⚬ Cyprus
  ⚬ Czechia
  ⚬ Denmark
  ⚬ Estonia
  ⚬ Finland
  ⚬ France
  ⚬ Germany
  ⚬ Greece
  ⚬ Hungary
  ⚬ Ireland
  ⚬ Italy
  ⚬ Latvia
  ⚬ Lithuania
  ⚬ Luxembourg
  ⚬ Malta
  ⚬ Netherlands
  ⚬ Norway
  ⚬ Poland
  ⚬ Portugal
  ⚬ Romania
  ⚬ Slovakia
  ⚬ Slovenia
  ⚬ Spain
  ⚬ Sweden
  ⚬ Switzerland
  ⚬ United Kingdom
  ⚬ Ukraine
  ⚬ Other:
  **Table:**
  Show/Hide Question
  Default > Hide Question
  **If Criteria** Q2c **is met show question**
4. Have you completed your residency training program? [FC]
  ⚬ Yes
  ⚬ No, my training is in progress
  **Table:**
  Skip Logic:	
  	Yes **If Selected, jump to** 8. [Q4a] 4a. Approximately how many months of brachytherapy training did you receive during your residency program?
  	No, my training is in progress **If Selected, jump to**
  	9. [Q4b.1] 4.b.1 Approximately how many months of brachytherapy training do you expect to receive during your program?
4a. Approximately how many months of brachytherapy training did you receive during your residency program? [PC]
  ⚬ None
  ⚬ 1 month
  ⚬ 2 months
  ⚬ 3 months
  ⚬ 4 months
  ⚬ 5 months
  ⚬ More than 5 months
  **Table:**
  Show/Hide Question
  Default > Hide Question
  **If Criteria** 4a **is met show question**
4b.1 Approximately how many months of brachytherapy training do you expect to receive during your program? [FC]
  ⚬ None
  ⚬ 1 months
  ⚬ 2 months
  ⚬ 3 months
  ⚬ 4 months
  ⚬ 5 months
  ⚬ More than 5 months
  **Table:**
  Show/Hide Question
  Default > Hide Question
  **If Criteria** 4b **is met show question**
4b.2 Approximately how many months of brachytherapy training have you received so far? [FC]
  ⚬ None
  ⚬ 1 months
  ⚬ 2 months
  ⚬ 3 months
  ⚬ 4 months
  ⚬ 5 months
  ⚬ More than 5 months
  **Table:**
  Skip Logic:
  None **If Selected, jump to** Thank you Page
  Show/Hide Question
  Default > Hide Question
  **If criteria** 4b **is met show question**
5. During which calendar year did your residency program begin? [FC]
  ⚬ 2017
  ⚬ 2018
  ⚬ 2019
  ⚬ 2020
  ⚬ 2021
  ⚬ Other:
6. Is (was) your brachytherapy training performed at your institution or is (was) your training performed at another institution? [FC]
  ⚬ In my institution only
  ⚬ Mainly in my institution, but partly in another institution
  ⚬ At another institution

### Training methods

7. What methods did your program use to teach didactic material for brachytherapy? Check all that apply. [FC]
  ⚬ Independent review of recommendations and regulations
  ⚬ Assigned reading without structured meetings
  ⚬ Assigned reading with structured meetings
  ⚬ AAPM virtual library presentation
  ⚬ Presentations from faculty/staff members
  ⚬ Presentations from residents
  ⚬ No didactic training
  ⚬ Other:
8. Which individual(s) typically perform brachytherapy treatment planning at your residency institution? Check all that apply? [FC]
  ⚬ Physician
  ⚬ Physicist
  ⚬ Dedicated brachytherapy dosimetrist
  ⚬ Dosimetrist
  ⚬ Medical physics resident
9. Which method(s) does your residency program use to teach brachytherapy treatment planning to medical physics residents? Check all that apply. [FC]
  ⚬ Observation
  ⚬ Physicist instruction
  ⚬ Dosimetrist instruction
  ⚬ Self‐learning by following written instructions or procedures
  ⚬ Self‐learning through review of previously created plans
  ⚬ Instruction from a co‐resident
  ⚬ Online or electronic resources
  ⚬ Other:

### Level of independence

10. How integrated/involved or separated/removed from the brachytherapy workflow did you feel while on your brachytherapy rotation(s)? [PC]
  ⚬ Very separated/removed
  ⚬ Somewhat separated/removed
  ⚬ Neutral
  ⚬ Somewhat integrated/involved
  ⚬ Very integrated/involved

The following questions ask about the number and types of cases (number of fractions) you participated in as a resident and your confidence level with each task.

Please indicate your confidence level in performing each of the tasks listed from (1)—Not confident at all to (5)—Very confident. Please select the number of cases (number of fractions) you were able to perform a specific task for a given procedure.

11. General [PC]
  **Table:**
  	**Confidence level**					**Number of cases**			
  	Not confident at all			Very confident					
  	1	2	3	4	5	0	≤5	>5	>10
  Daily quality assurance of remote afterloader	◯	◯	◯	◯	◯	◯	◯	◯	◯
  Source exchange quality assurance of remote afterloader	◯	◯	◯	◯	◯	◯	◯	◯	◯
  Assay of loose seeds	◯	◯	◯	◯	◯	◯	◯	◯	◯
  Connection of patient to afterloader	◯	◯	◯	◯	◯	◯	◯	◯	◯
  Pre‐/post‐treatment surveys	◯	◯	◯	◯	◯	◯	◯	◯	◯
  Applicator quality assurance	◯	◯	◯	◯	◯	◯	◯	◯	◯
12. Please select the types of brachytherapy procedures performed at your residency institution. Check all that apply. [PC]
  ⚬ Low dose rate (LDR)
  ⚬ High dose rate/Pulsed Dose Rate (HDR/PDR)
  ⚬ Intraoperative Radiation Therapy (IORT)
  ⚬ TransArterial Radioembolization (TARE, e.g., ^90^Y microspheres)
  ⚬ Radiopharmaceuticals (^177^Lu, ^223^Ra, etc.)

#### Low Dose Rate (LDR)

The following questions refer to LDR procedures.

Please indicate your confidence level in performing each of the tasks listed from (1)—Not confident at all to (5)—Very confident. Please select the number of times you were able to perform a specific task for a given procedure.

Check N/A only if your institution does not offer this procedure/technique/task.

**Ocular Eye—Plaques**

**Table:**

	**Confidence level**					**Number of cases**				**N/A**
	Not confident at all			Very confident						
	1	2	3	4	5	0	≤5	>5	>10	
Treatment planning	◯	◯	◯	◯	◯	◯	◯	◯	◯	◯
Treatment plan check	◯	◯	◯	◯	◯	◯	◯	◯	◯	◯
Treatment/procedure	◯	◯	◯	◯	◯	◯	◯	◯	◯	◯

**Prostate**

**Table:**

	**Confidence level**					**Number of cases**				**N/A**
	Not confident at all			Very confident						
	1	2	3	4	5	0	≤5	>5	>10	
Treatment planning	◯	◯	◯	◯	◯	◯	◯	◯	◯	◯
Treatment plan check	◯	◯	◯	◯	◯	◯	◯	◯	◯	◯
Treatment/procedure	◯	◯	◯	◯	◯	◯	◯	◯	◯	◯

**Brain**

**Table:**

	**Confidence level**					**Number of cases**				**N/A**
	Not confident at all			Very confident						
	1	2	3	4	5	0	≤5	>5	>10	
Treatment planning	◯	◯	◯	◯	◯	◯	◯	◯	◯	◯
Treatment plan check	◯	◯	◯	◯	◯	◯	◯	◯	◯	◯
Treatment/procedure	◯	◯	◯	◯	◯	◯	◯	◯	◯	◯

**Interstitial—Other**

**Table:**

	**Confidence level**					**Number of cases**				**N/A**
	Not confident at all			Very confident						
	1	2	3	4	5	0	≤5	>5	>10	
Treatment planning	◯	◯	◯	◯	◯	◯	◯	◯	◯	◯
Treatment plan check	◯	◯	◯	◯	◯	◯	◯	◯	◯	◯
Treatment/procedure	◯	◯	◯	◯	◯	◯	◯	◯	◯	◯

#### High Dose Rate/Pulsed Dose Rate (HDR/PDR)

The following questions refer to HDR/PDR procedures.

Please indicate your confidence level in performing each of the tasks listed from (1)—Not confident at all to (5)—Very confident. Please select the number of times you were able to perform a specific task for a given procedure.

Check N/A only if your institution does not offer this procedure/technique/task.

**Prostate**

**Table:**

	**Confidence level**					**Number of cases**				**N/A**
	Not confident at all			Very confident						
	1	2	3	4	5	0	≤5	>5	>10	
Treatment planning	◯	◯	◯	◯	◯	◯	◯	◯	◯	◯
Treatment plan check	◯	◯	◯	◯	◯	◯	◯	◯	◯	◯
Procedure	◯	◯	◯	◯	◯	◯	◯	◯	◯	◯

**Gynecological**

**Table:**

	**Confidence level**					**Number of cases**				**N/A**
	Not confident at all			Very confident						
	1	2	3	4	5	0	≤5	>5	>10	
Treatment planning	◯	◯	◯	◯	◯	◯	◯	◯	◯	◯
Treatment plan check	◯	◯	◯	◯	◯	◯	◯	◯	◯	◯
Procedure	◯	◯	◯	◯	◯	◯	◯	◯	◯	◯

**Endobronchial**

**Table:**

	**Confidence level**					**Number of cases**				**N/A**
	Not confident at all			Very confident						
	1	2	3	4	5	0	≤5	>5	>10	
Treatment planning	◯	◯	◯	◯	◯	◯	◯	◯	◯	◯
Treatment plan check	◯	◯	◯	◯	◯	◯	◯	◯	◯	◯
Procedure	◯	◯	◯	◯	◯	◯	◯	◯	◯	◯

**Skin/Surface**

**Table:**

	**Confidence level**					**Number of cases**				**N/A**
	Not confident at all			Very confident						
	1	2	3	4	5	0	≤5	>5	>10	
Treatment planning	◯	◯	◯	◯	◯	◯	◯	◯	◯	◯
Treatment plan check	◯	◯	◯	◯	◯	◯	◯	◯	◯	◯
Procedure	◯	◯	◯	◯	◯	◯	◯	◯	◯	◯

**Breast**

**Table:**

	**Confidence level**					**Number of cases**				**N/A**
	Not confident at all			Very confident						
	1	2	3	4	5	0	≤5	>5	>10	
Treatment planning	◯	◯	◯	◯	◯	◯	◯	◯	◯	◯
Treatment plan check	◯	◯	◯	◯	◯	◯	◯	◯	◯	◯
Procedure	◯	◯	◯	◯	◯	◯	◯	◯	◯	◯

**Interstitial—Other**

**Table:**

	**Confidence level**					**Number of cases**				**N/A**
	Not confident at all			Very confident						
	1	2	3	4	5	0	≤5	>5	>10	
Treatment planning	◯	◯	◯	◯	◯	◯	◯	◯	◯	◯
Treatment plan check	◯	◯	◯	◯	◯	◯	◯	◯	◯	◯
Procedure	◯	◯	◯	◯	◯	◯	◯	◯	◯	◯

#### Intraoperative Radiation Therapy (IORT)

The following questions refer to IORT procedures.

Please indicate your confidence level in performing each of the tasks listed from (1)—Not confident at all to (5)—Very confident. Please select the number of times you were able to perform a specific task for a given procedure.

Check N/A only if your institution does not offer this procedure/technique/task.

**Breast**

**Table:**

	**Confidence level**					**Number of cases**				**N/A**
	Not confident at all			Very confident						
	1	2	3	4	5	0	≤5	>5	>10	
Treatment planning	◯	◯	◯	◯	◯	◯	◯	◯	◯	◯
Treatment plan check	◯	◯	◯	◯	◯	◯	◯	◯	◯	◯
Procedure	◯	◯	◯	◯	◯	◯	◯	◯	◯	◯

**Gastrointestinal**

**Table:**

	**Confidence level**					**Number of cases**				**N/A**
	Not confident at all			Very confident						
	1	2	3	4	5	0	≤5	>5	>10	
Treatment planning	◯	◯	◯	◯	◯	◯	◯	◯	◯	◯
Treatment plan check	◯	◯	◯	◯	◯	◯	◯	◯	◯	◯
Procedure	◯	◯	◯	◯	◯	◯	◯	◯	◯	◯

#### TransArterial Radioembolization (TARE)

The following questions refer to TARE procedures.

Please indicate your confidence level in performing each of the tasks listed from (1)—Not confident at all to (5)—Very confident. Please select the number of times you were able to perform a specific task for a given procedure.

Check N/A only if your institution does not offer this procedure/technique/task.

**Any Site**

**Table:**

	**Confidence level**					**Number of cases**				**N/A**
	Not confident at all			Very confident						
	1	2	3	4	5	0	≤5	>5	>10	
Treatment planning	◯	◯	◯	◯	◯	◯	◯	◯	◯	◯
Treatment plan check	◯	◯	◯	◯	◯	◯	◯	◯	◯	◯
Procedure	◯	◯	◯	◯	◯	◯	◯	◯	◯	◯

#### Radiopharmaceuticals

The following questions refer to radiopharmaceutical procedures.

Please indicate your confidence level in performing each of the tasks listed from (1)—Not confident at all to (5)—Very confident. Please select the number of times you were able to perform a specific task for a given procedure.

Check N/A only if your institution does not offer this procedure/technique/task.

**Any Site**

**Table:**

	**Confidence level**					**Number of cases**				**N/A**
	Not confident at all			Very confident						
	1	2	3	4	5	0	≤5	>5	>10	
Treatment planning	◯	◯	◯	◯	◯	◯	◯	◯	◯	◯
Treatment plan check	◯	◯	◯	◯	◯	◯	◯	◯	◯	◯
Procedure	◯	◯	◯	◯	◯	◯	◯	◯	◯	◯

13. Do you believe you gained enough training as a resident to practice brachytherapy confidently at the completion of your training program? [PC]
  ⚬ Yes—for all sites treated at my current institution
  ⚬ Yes—for some sites treated at my current institution
  ⚬ No
  **Table:**
  Skip Logic:	
  	Yes—for all sites treated at my current institution **If Selected, jump to**
  	49. [Q14] 14. Which one of the following factors would most help improve your level of confidence? Yes—for some sites treated at my current institution **If Selected, jump to**
  	48. [Q13b‐c] 13.b‐c. If you did not achieve your desired level of confidence during training, which factors do you believe may have contributed to this outcome? Select all that apply.
  	No **If Selected, jump to**
  	48. [Q13b‐c] 13.b‐c. If you did not achieve your desired level of confidence during training, which factors do you believe may have contributed to tis outcome? Select all that apply.
13.b‐c. If you did not achieve your desired level of confidence during training, which factors do you believe contributed to this outcome? *Select all that apply*. [PC]
  ⚬ N/A—I feel very comfortable performing brachytherapy procedures independently
  ⚬ Interest level in topic
  ⚬ Rotation time constraints (not enough time to learn)
  ⚬ Clinical time constraints (pace of procedures)
  ⚬ Hesitancy of supervisor to provide adequate independence
  ⚬ Departmental policy (interpretation of state/federal regulations)
  ⚬ Regulations that prohibit trainee independence
  ⚬ Clinical environment
  ⚬ Caseload—too low
  ⚬ Other:
  **Table:**
  Show/Hide Question
  Default > Hide Question
  **If Criteria** Q13b‐c **is met show question**
14. Which one of the following factors would most help improve your level of confidence? [PC]
  ⚬ N/A—I feel very comfortable performing brachytherapy procedures independently
  ⚬ Increased length of rotation
  ⚬ Increased caseload
  ⚬ Increased involvement in cases
  ⚬ Additional didactic training

### Future plans

15. How did your level of interest in performing brachytherapy procedures change after completing your brachytherapy training? [PC]
  ⚬ Increased considerably
  ⚬ Increased some
  ⚬ Did not change
  ⚬ Decreased some
  ⚬ Decreased considerably
16. Do you want to perform brachytherapy procedures at your next position? [FC]
  ⚬ Yes
  ⚬ No
  ⚬ Unsure
17. If you already have your next position arranged or have started in your first employed position, will you do you perform brachytherapy procedures? [FC]
  ⚬ Yes
  ⚬ No
  ⚬ Unsure
  ⚬ N/A—I do not yet have my next position arranged

### Additional training opportunities

18. Do you wish you had more training opportunities in brachytherapy? [PC]
  ⚬ Yes
  ⚬ No
  ⚬ Unsure
  **Table:**
  Skip Logic:	
  	Yes, **If Selected, jump to**
  	56. [Q19] 19. Please indicate your level of likelihood with the following: If your residency program had an elective rotation, how likely would you be to select brachytherapy as the elective topic during your training?
  	No **If Selected, jump to** 59. [BPI] Brachytherapy Practice Impressions
  	Unsure **If Selected, jump to**
  	56. [Q19] 19. Please indicate your level of likelihood with the following: If your residency program had an elective rotation, how likely would you be to select brachytherapy as the elective topic during your training?
19. Please indicate your level of likelihood with the following: If your residency program had an elective rotation, how likely would you be to select brachytherapy as the elective topic during your training? [FC]
  **Table:**
  Very unlikely				Very likely
  1	2	3	4	5
  ◯	◯	◯	◯	◯
  **Table:**
  Show/Hide Question
  Default > Hide Question
  **If Criteria** Q18ac **is met show question**
20. Would you be interested (or have been interested) in performing a brachytherapy rotation at an institution with a higher caseload and/or with a greater variety of brachytherapy treatments than your training institution? [FC]
  ⚬ Yes
  ⚬ No
  ⚬ Unsure
  **Table:**
  Show/Hide Question
  Default > Hide Question
  **If Criteria** Q18ac **is met show question**
21. If offered, would you be interested in pursuing a fellowship in brachytherapy following your residency training? If you have already started your first position post‐training, would you have been interested in pursuing a fellowship in brachytherapy following your residency? [FC]
  ⚬ No, regardless of length of fellowship training
  ⚬ Yes, if fellowship is less than 1 month long
  ⚬ Yes, if fellowship is less than 6 months long
  ⚬ Yes, if fellowship is 6 to 12 months long
  **Table:**
  Show/Hide Question
  Default > Hide Question
  **If Criteria** Q18ac **is met show question**

### Brachytherapy practice impressions

22. Do you believe your residency program director values (valued) your education in the area of brachytherapy? [FC]
  ⚬ Yes
  ⚬ No
  ⚬ Unsure
23. How do you think the clinical relevance of brachytherapy will change in the next 10 years for the following treatment sites? [FC]
  **Table:**
  	Increase	Stay the same	Decrease
  Breast	◯	◯	◯
  Gynecological (GYN)	◯	◯	◯
  Prostate	◯	◯	◯
  Ocular	◯	◯	◯
  Endobronch	◯	◯	◯
  Skin/Surface	◯	◯	◯
  Brain	◯	◯	◯
  Gastrointestinal (GI)	◯	◯	◯
  Liver	◯	◯	◯
  Metastases	◯	◯	◯
  Other	◯	◯	◯
24. Please indicate level of importance: How important do you believe it is for medical physics residents to learn brachytherapy? [FC]
  **Table:**
  Not important at all				Very important
  1	2	3	4	5
  ◯	◯	◯	◯	◯
25. Please indicate your level of enjoyment: How much did you enjoy your brachytherapy rotation compared to other rotations? [FC]
  **Table:**
  I enjoyed it significantly less			I enjoyed it more than my other rotations	
  1	2	3	4	5
  ◯	◯	◯	◯	◯
26. Is there additional information that you would like to provide regarding the brachytherapy component of your residency? [FC]
