# Supplementary material for: AAPM BTSC Report 377.B: Physicist brachytherapy training in 2022 – A survey of therapeutic medical physics residents
Source: J Appl Clin Med Phys. 2024 Oct 7;25(11):e14501. doi: 10.1002/acm2.14501 (PMC11539968; doi:10.1002/acm2.14501)
Supplement: Supplementary file 1 — Supporting information [file ACM2-25-e14501-s001.docx]

**Appendix B**

| 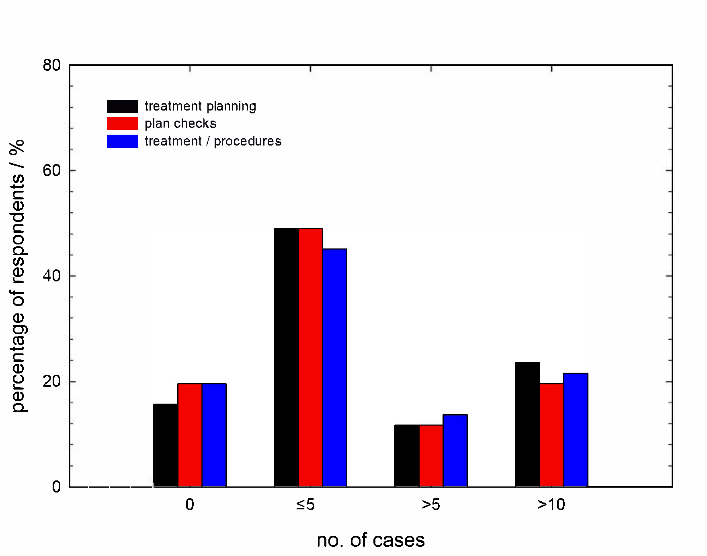 | 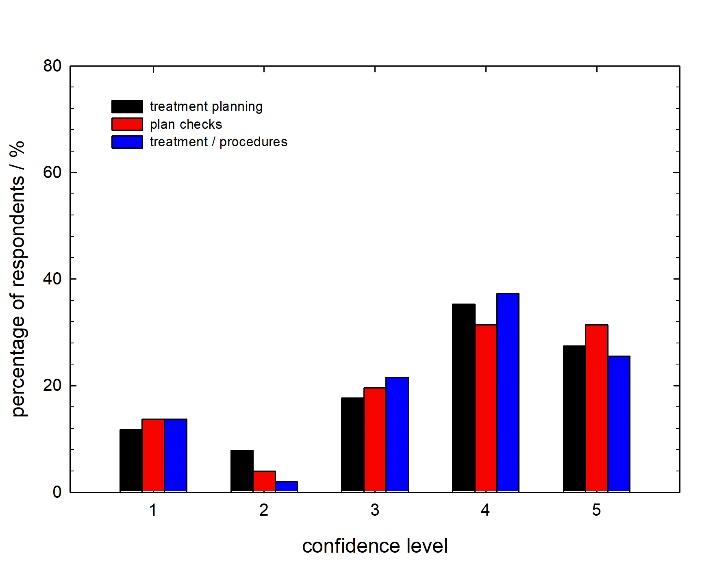 |
| --- | --- |
| (a) | (b) |
| 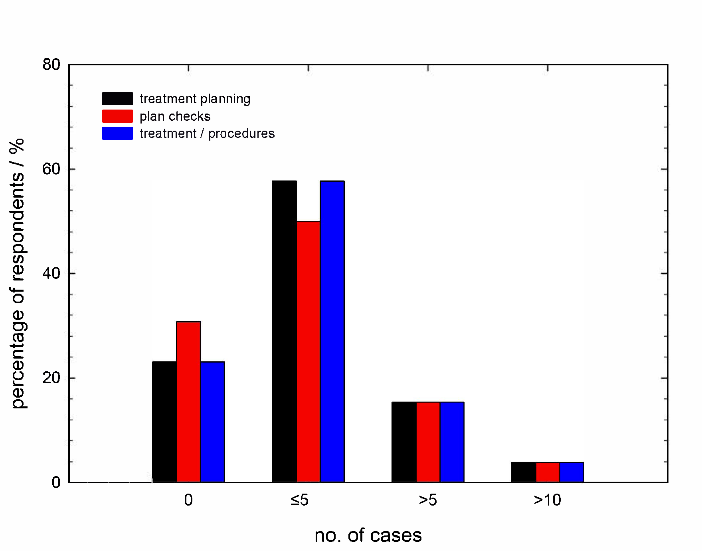 | 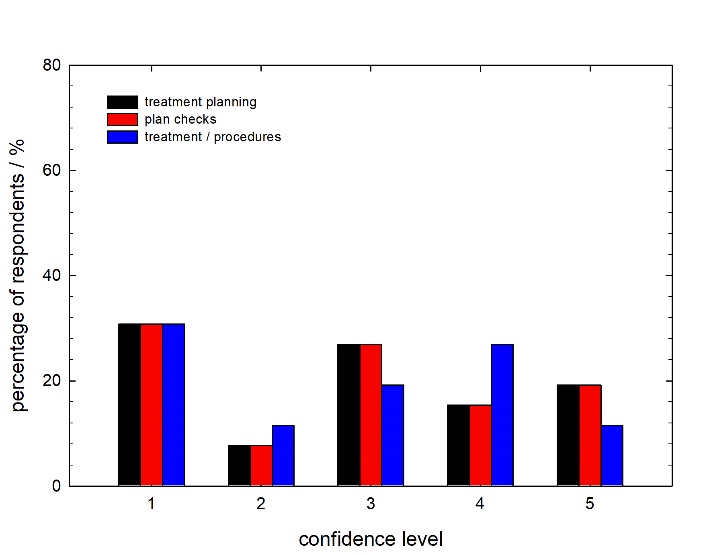 |
| (c) | (d) |
| 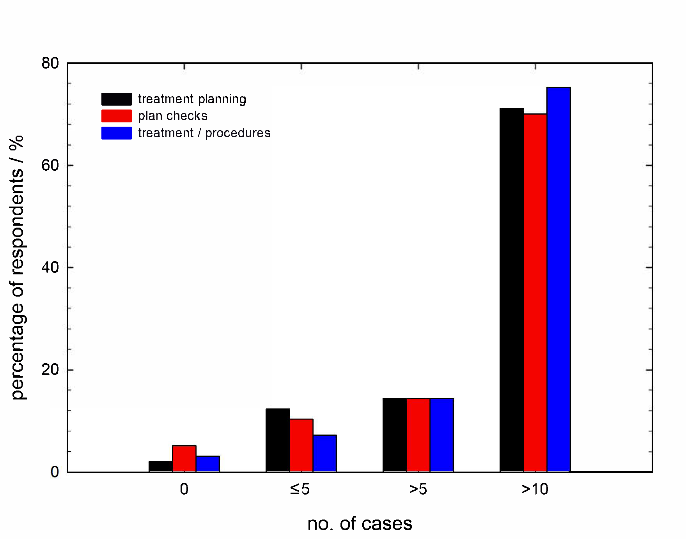 | 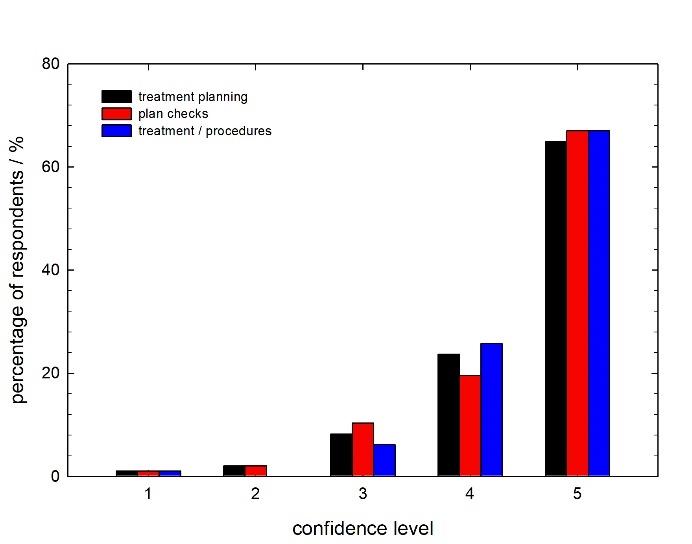 |
| (e) | (f) |
| 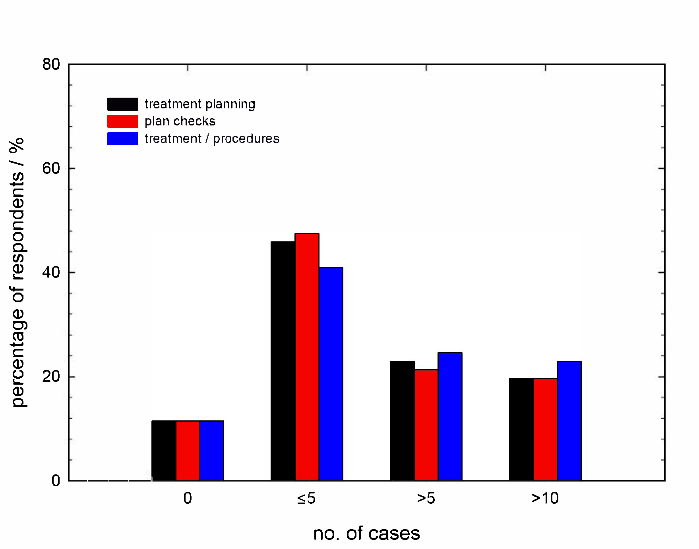 | 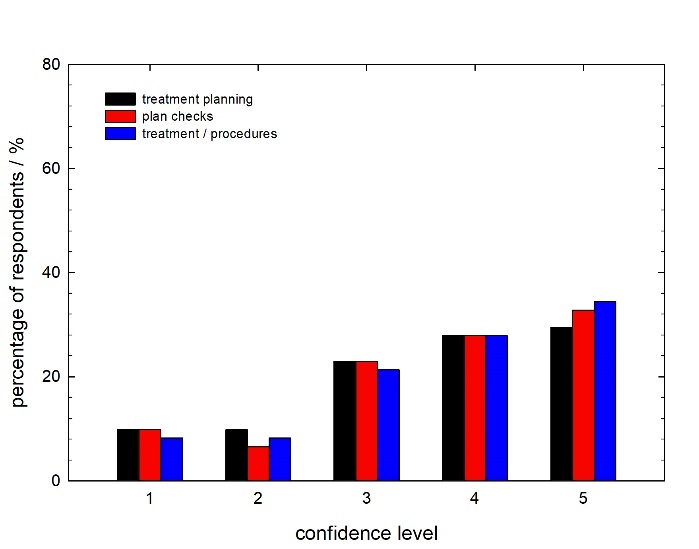 |
| (g) | (h) |
| 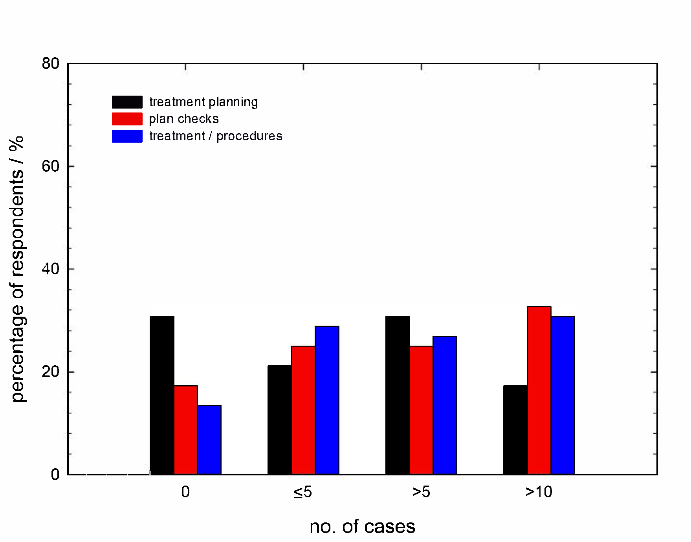 | 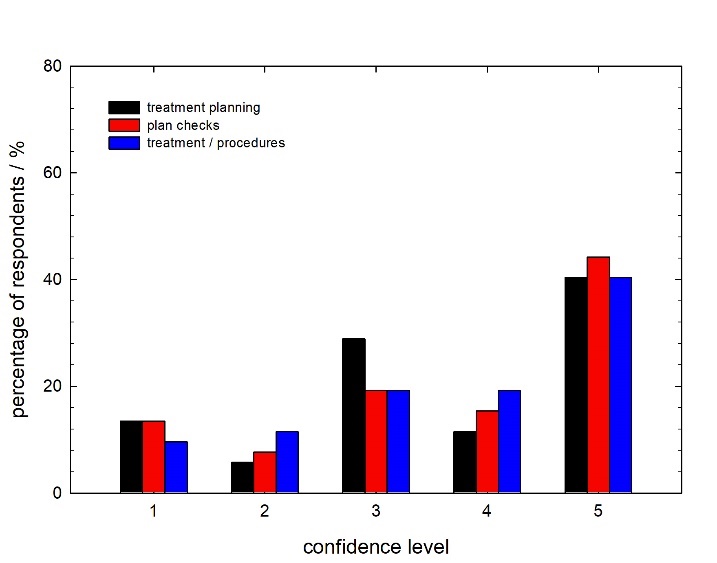 |
| (i) | (j) |
| 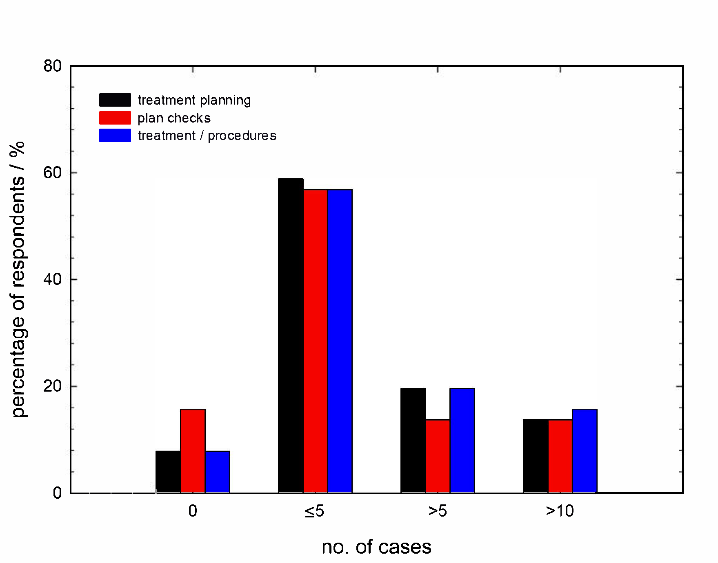 | 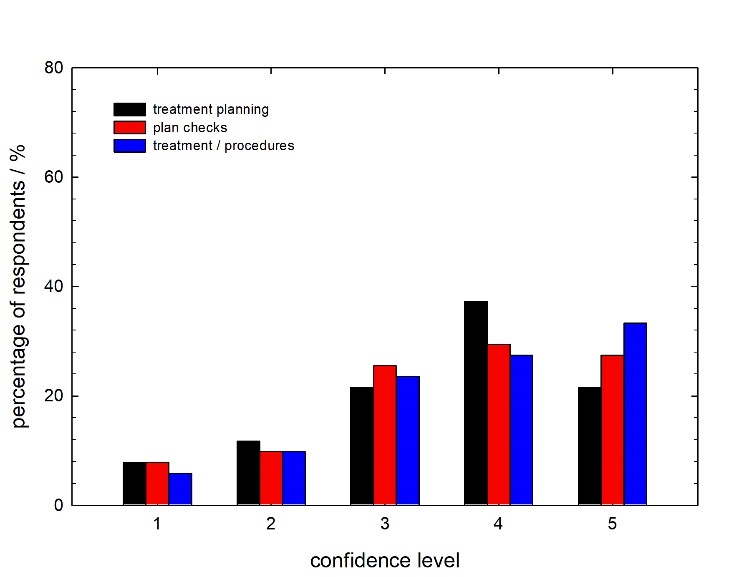 |
| (k) | (l) |
| 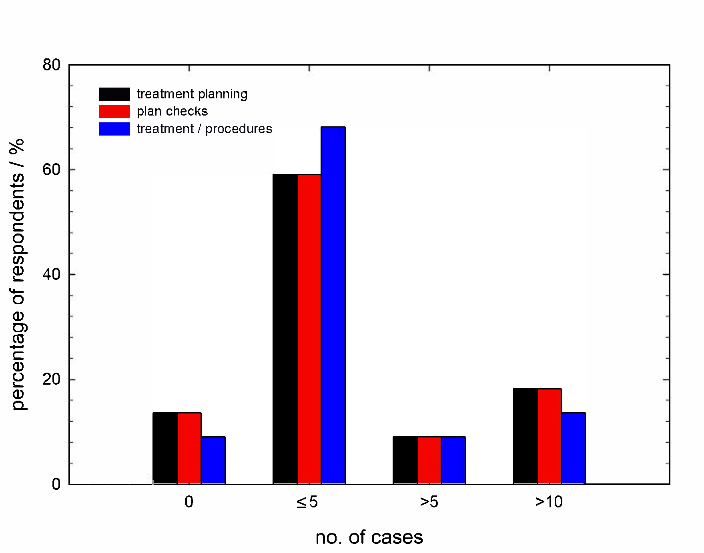 | 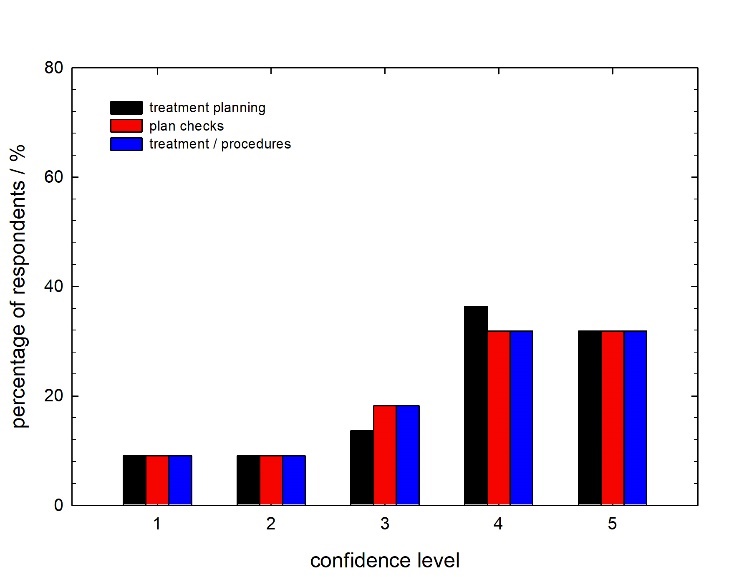 |
| (m) | (n) |
| 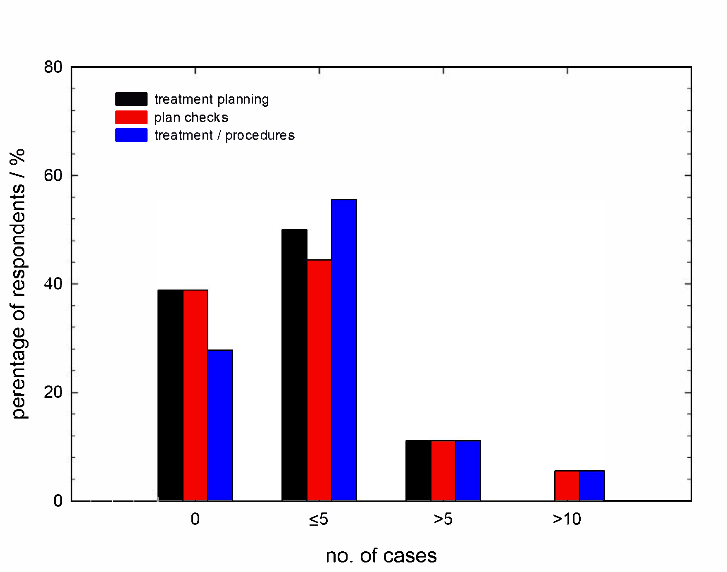 | 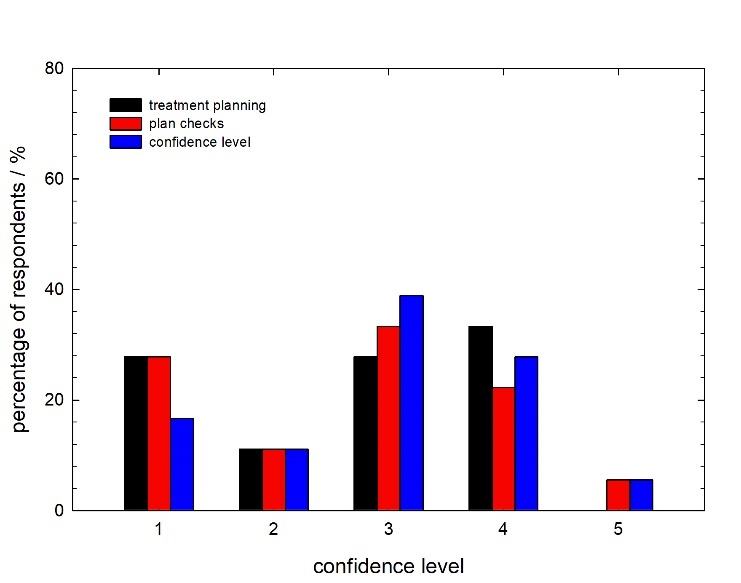 |
| (o) | (p) |
| 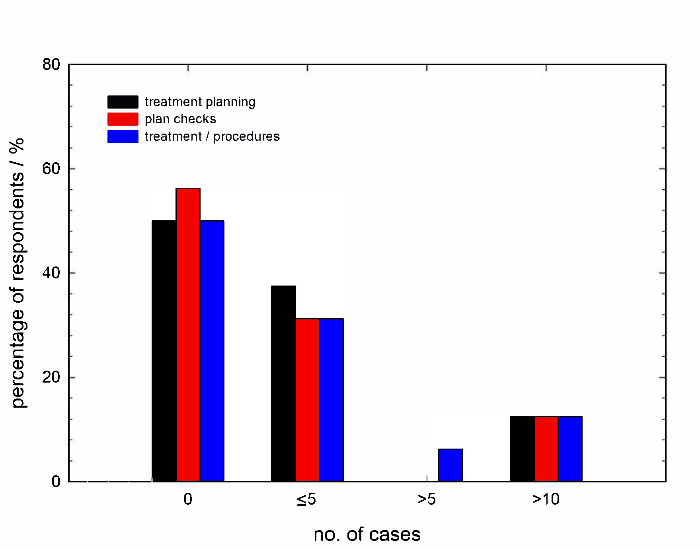 | 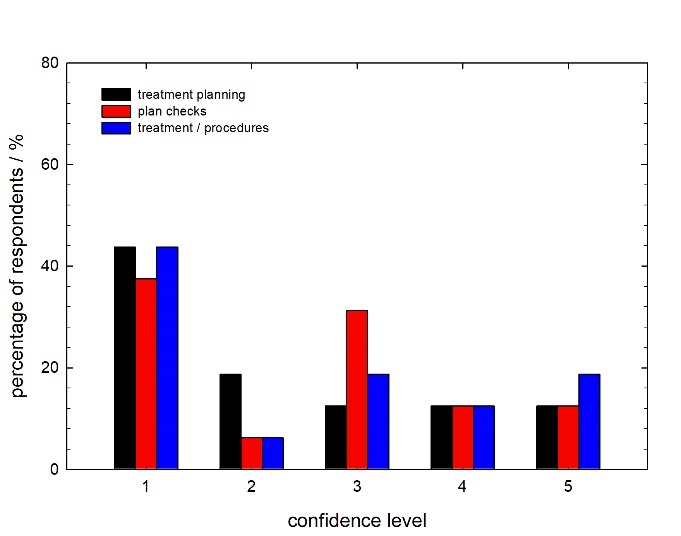 |
| (q) | (r) |
| 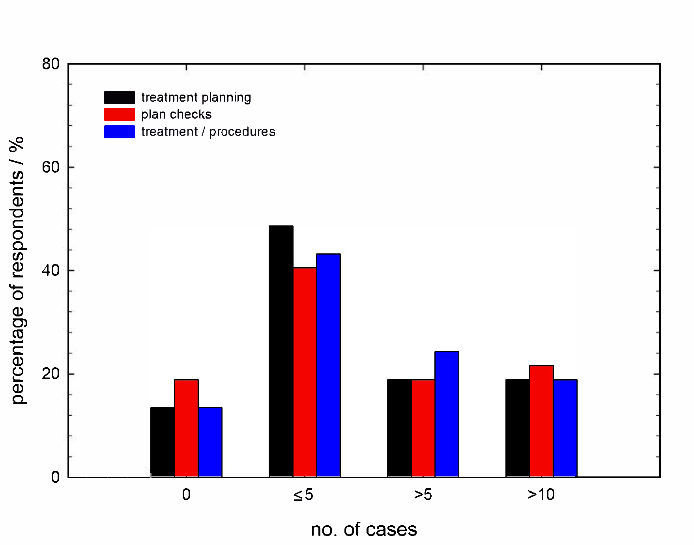 | 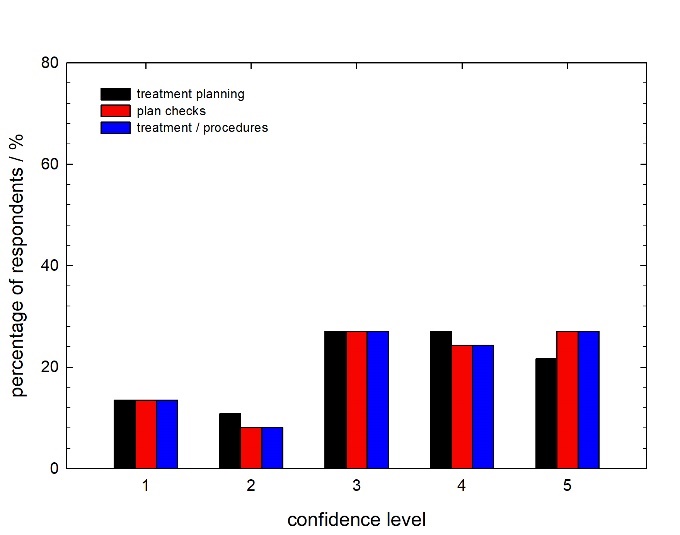 |
| (s) | (t) |
| 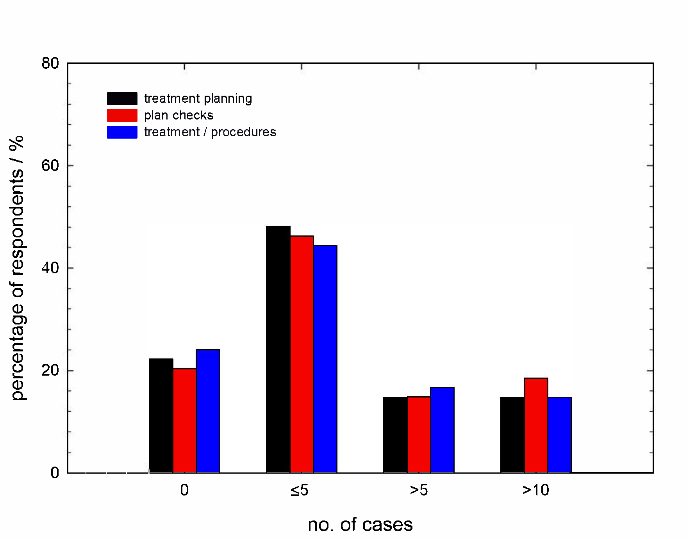 | 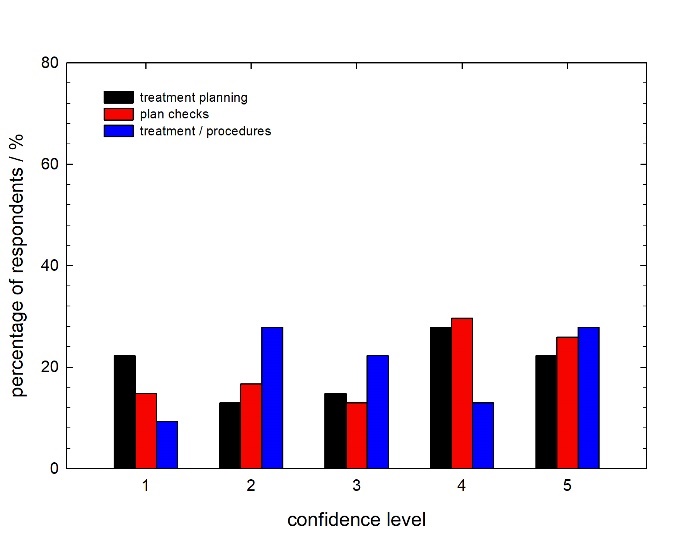 |
| (u) | (v) |
| 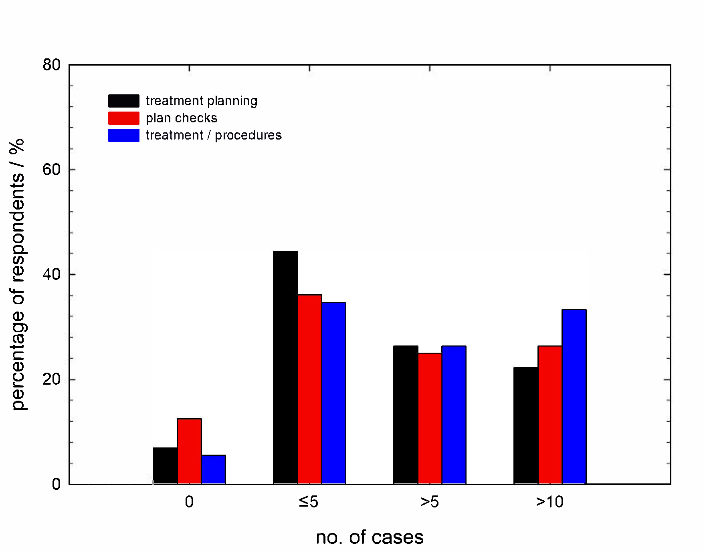 | 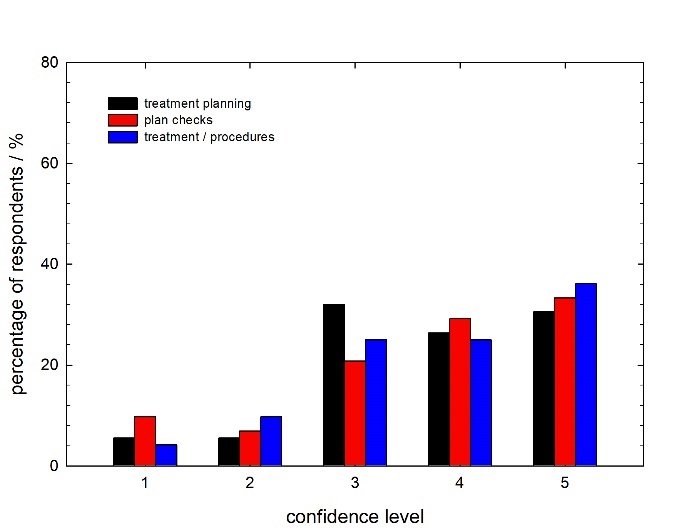 |
| (w) | (x) |
| 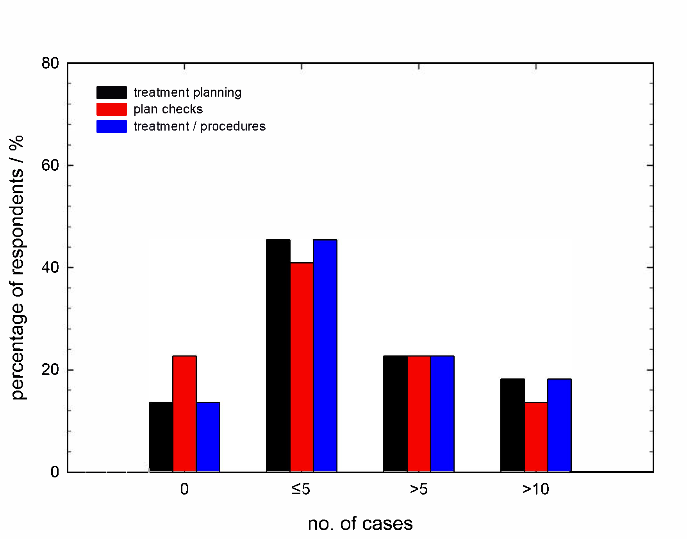 | 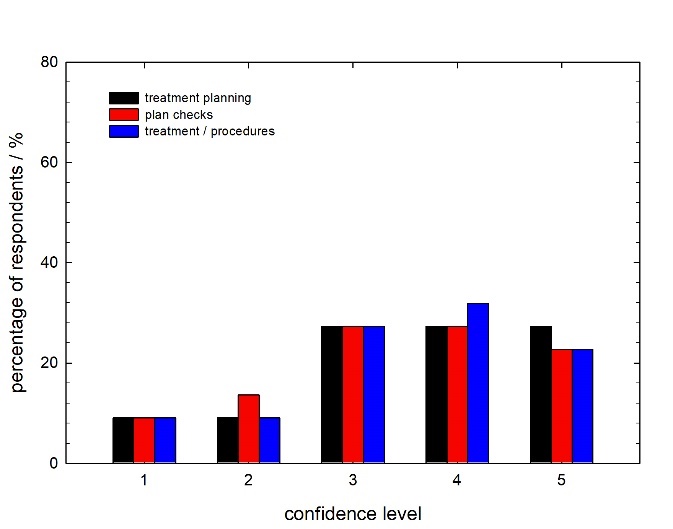 |
| (y) | (z) |
| 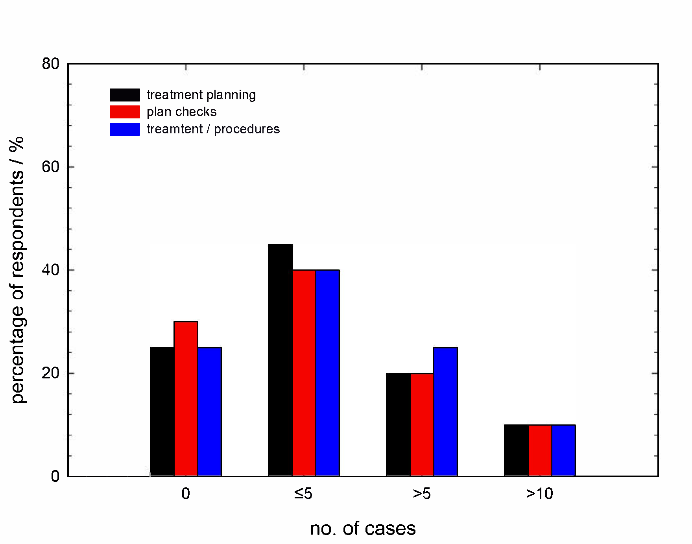 | 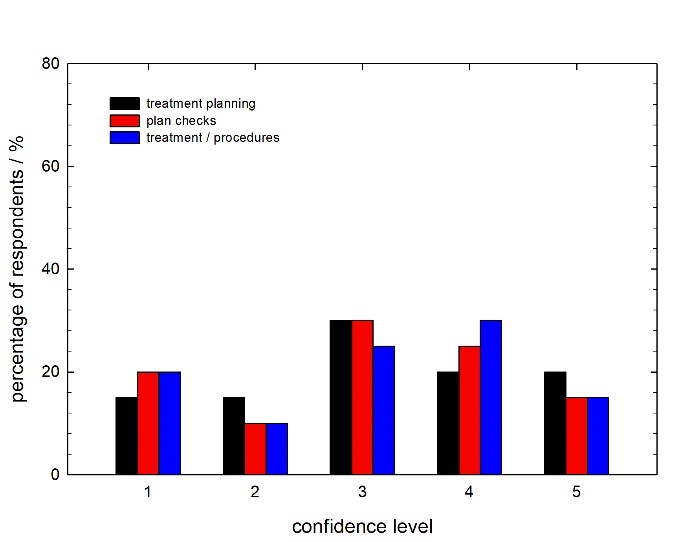 |
| (aa) | (bb) |

Figure B.1: Caseload and confidence data presented for the fourteen modality and disease site combinations investigated. Data was collected for areas of treatment planning, plan checks, and treatment procedures. Data in each plot is presented as percentages normalized to the number of respondents who indicated training was received for a specific combination of treatment modality and disease site. Results include case load and confidence data for HDR breast (a and b), HDR endometrial (c and d), HDR GYN (e and f), HDR interstitial (g and h), HDR prostate (i and j), HDR skin (k and l), IORT breast (m and n), IORT GI (o and p), LDR brain (q and r), LDR interstitial (s and t), LDR ocular (u and v), LDR prostate (w and x), radiopharmaceuticals (y and z), and TARE (aa and bb).

Table B.1: Statistical analysis for Question 12 stratified by individual brachytherapy clinical procedures and competencies. A Spearman correlation coefficient was calculated for each reported category as well as the median and mode confidence level and case load values.

| **Category** | **Spearman Rho coefficient** | **Median Confidence level** | **Mode Confidence Level** | **Median Caseload** | **Mode Caseload** |
| --- | --- | --- | --- | --- | --- |
| **Low Dose Rate Ocular** |  |  |  |  |  |
| Treatment Planning | 0.8584 | 3 | 1 | <=5 | 0 |
| Treatment Plan Check | 0.8682 | 3 | 1 | <=5 | 0 |
| Treatment / Procedure | 0.8470 | 3 | 1 | <=5 | 0 |
| **Low Dose Rate Prostate** |  |  |  |  |  |
| Treatment Planning | 0.8541 | 4 | 3 | <=5 | <=5 |
| Treatment Plan Check | 0.8461 | 4 | 5 | <=5 | <=5 |
| Treatment / Procedure | 0.8523 | 4 | 5 | >5 | <=5 |
| **Low Dose Rate Brain** |  |  |  |  |  |
| Treatment Planning | 0.9380 | 1 | 1 | 0 | 0 |
| Treatment Plan Check | 0.8815 | 1 | 1 | 0 | 0 |
| Treatment / Procedure | 0.9229 | 1 | 1 | 0 | 0 |
| **Low Dose Rate Interstitial Other** |  |  |  |  |  |
| Treatment Planning | 0.8964 | 2 | 1 | 0 and <=5 | 0 |
| Treatment Plan Check | 0.9154 | 2 | 1 | 0 | 0 |
| Treatment / Procedure | 0.8979 | 2 | 1 | 0 and <=5 | 0 |
| **High Dose Rate Prostate** |  |  |  |  |  |
| Treatment Planning | 0.9111 | 3 | 5 | <=5 | 0 |
| Treatment Plan Check | 0.8996 | 3 | 5 | <=5 | 0 |
| Treatment / Procedure | 0.8770 | 3 | 5 | <=5 | 0 |
| **High Dose Rate Gyn** |  |  |  |  |  |
| Treatment Planning | 0.7231 | 5 | 5 | >10 | >10 |
| Treatment Plan Check | 0.7900 | 5 | 5 | >10 | >10 |
| Treatment / Procedure | 0.7523 | 5 | 5 | >10 | >10 |
| **High Dose Rate Endobronchial** |  |  |  |  |  |
| Treatment Planning | 0.8684 | 2 | 1 | <=5 | 0 |
| Treatment Plan Check | 0.8253 | 2 | 1 | 0 and <=5 | 0 |
| Treatment / Procedure | 0.8272 | 2 | 1 | <=5 | 0 |
| **High Dose Rate Skin** |  |  |  |  |  |
| Treatment Planning | 0.8202 | 3 | 4 | <=5 | <=5 |
| Treatment Plan Check | 0.7920 | 3 | 1 | <=5 | <=5 |
| Treatment / Procedure | 0.8272 | 3 | 5 | <=5 | <=5 |
| **High Dose Rate Breast** |  |  |  |  |  |
| Treatment Planning | 0.8068 | 3 | 4 | <=5 | <=5 |
| Treatment Plan Check | 0.7926 | 3 | 1 | <=5 | <=5 |
| Treatment / Procedure | 0.8397 | 3 | 1 | <=5 | <=5 |
| **High Dose Rate Interstitial** |  |  |  |  |  |
| Treatment Planning | 0.8267 | 4 | 5 | <=5 | <=5 |
| Treatment Plan Check | 0.7963 | 4 | 5 | <=5 | <=5 |
| Treatment / Procedure | 0.8374 | 4 | 5 | <=5 | <=5 |
| **IORT Breast** |  |  |  |  |  |
| Treatment Planning | 0.8613 | 3 | 1 | <=5 | <=5 |
| Treatment Plan Check | 0.8452 | 3 | 1 | <=5 | <=5 |
| Treatment / Procedure | 0.8188 | 3 | 3 | <=5 | <=5 |
| **IORT GI** |  |  |  |  |  |
| Treatment Planning | 0.8638 | 3 | 1 | <=5 | <=5 |
| Treatment Plan Check | 0.8892 | 3 | 1 | <=5 | <=5 |
| Treatment / Procedure | 0.8677 | 3 | 1 | <=5 | <=5 |
| **TARE** |  |  |  |  |  |
| Treatment Planning | 0.8608 | 3 | 1 | <=5 | <=5 |
| Treatment Plan Check | 0.8563 | 3 | 1 | <=5 | 0 |
| Treatment / Procedure | 0.8272 | 3 | 1 | <=5 | <=5 |
| **Radiopharmaceutical** |  |  |  |  |  |
| Treatment Planning | 0.7768 | 3 | 3 | <=5 | <=5 |
| Treatment Plan Check | 0.7691 | 3 | 3 | <=5 | <=5 |
| Treatment / Procedure | 0.7360 | 4 | 4 | <=5 | <=5 |
